# Fission yeast Whi5 represses MBF-dependent transcription in quiescent cells

**DOI:** 10.1016/j.isci.2025.114576

**Published:** 2025-12-30

**Authors:** Celia Gálvez-Merchán, Rafael López-San Segundo, M. Belén Suárez, Daniel González-Álvarez, José Ayté, Livia Pérez-Hidalgo, Sergio Moreno

**Affiliations:** 1 Institute of Functional Biology and Genomics, CSIC, University of Salamanca, 37007 Salamanca, Spain; 2 Institute of Functional Biology and Genomics, University of Salamanca, CSIC, 37007 Salamanca, Spain; 3 Departament of Microbiology and Genetics, University of Salamanca, 37007 Salamanca, Spain; 4 Oxidative Stress and Cell Cycle Group, Universitat Pompeu Fabra, 08003 Barcelona, Spain

**Keywords:** Natural sciences, Biological sciences, Molecular biology

## Abstract

When cells arrest in G1 to enter quiescence, the transcriptional machinery that drives the G1/S transition must be inactivated. In budding yeast and mammals, this repression is mediated by the Whi5 and Retinoblastoma (Rb) proteins, which inhibit the SBF and E2F transcription factors, respectively. In fission yeast, the MBF complex is functionally analogous to SBF and E2F, and Whi5/Mug54 has been predicted to act as a G1/S transcriptional repressor. Here, we show that upon nitrogen starvation, Whi5 accumulates in the nucleus and is required to repress MBF-dependent genes during quiescence. Mass spectrometry and bimolecular fluorescence complementation (BiFC) demonstrate that Whi5 physically associates with components of both the MBF complex and the histone deacetylase Clr6-I complex. Moreover, Whi5 is required for the interaction between MBF and Clr6-I, supporting a model in which Whi5 represses MBF-dependent genes in quiescent cells by recruiting HDAC activity to their promoters.

## Highlights

• Fission yeast Whi5 is a repressor of MBF-dependent transcription in quiescent cells
• Under nitrogen starvation, Whi5 localizes to the nucleus and interacts with MBF
• Whi5 represses MBF-dependent gene expression by recruiting the HDAC complex, Clr6-I

## Graphical abstract

**Figure:**
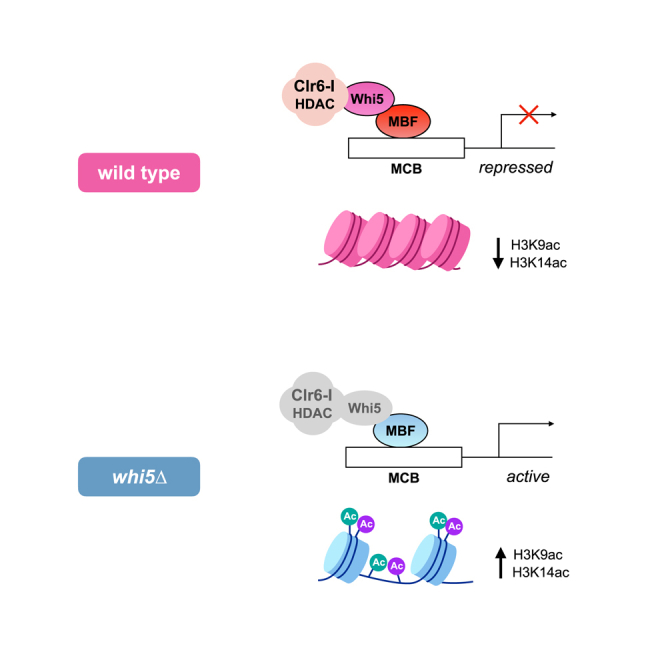

## Introduction

In the absence of nitrogen, fission yeast cells undergo two rounds of cell division without cell growth and arrest in the G1 phase of the cell cycle. Depending on the presence or not of a mating partner, fission yeast cells may undergo meiosis and sporulation or enter a reversible dormant state known as quiescence, respectively. Both differentiation processes involve a profound change in the transcriptional program. In meiosis, changes in transcription induce cells to undergo a premeiotic S-phase, followed by two meiotic divisions and spore formation.^1^ In the case of quiescence, although cells are transcriptionally and metabolically active, a global transcription shut off affects a large part of the genome.^2^^,^^3^^,^^4^^,^^5^^,^^6^^,^^7^^,^^8^^,^^9^ Switching off the genes involved in proliferation is critical because, if defective, it can lead to untimely re-entry into the cell cycle, which in yeast cells can affect survival and, in the case of human cells, can lead to pathologies such as cancer.^2^^,^^10^^,^^11^^,^^12^ Among the chromatin modifiers involved in the establishment and maintenance of quiescence, histone deacetylases (HDACs) play an important role in budding yeast.^4^^,^^13^ However, in fission yeast, heterochromatin establishment in quiescence has been shown to depend on RNAi and histone methylation.^2^^,^^5^^,^^6^

Transcriptional waves regulate major transitions between cell cycle phases. Gene expression at the G1/S transition depends on the transcription factor complexes MluI cell cycle box-binding factor (MBF), and Swi4/Swi6 cell cycle box-binding factor (SBF) in budding yeast and E2F-DP in mammals.^14^ In the fission yeast *Schizosaccharomyces pombe*, the G1/S transcriptional wave is controlled by the MBF complex, which, in vegetative cells, is composed of the DNA-binding Res1-Res2 heterodimer and Cdc10, an essential component of the complex.^15^^,^^16^^,^^17^ In addition, several proteins interact with this core complex, modulating its activity during the cell cycle. Co-activator Rep2, which binds MBF throughout the cell cycle, acts as a hub, facilitating the MBF-dependent transcription through the recruitment of the transcriptional co-activator complex SAGA (Spt-Ada-Gcn5 acetyltransferase) and the chromatin remodelers RSC and SWI/SNF complexes.^18^^,^^19^^,^^20^ The Ino80 complex physically interacts with Cdc10 and is required to fully and timely activate MBF-dependent transcription.^21^ The co-repressors Nrm1 and Yox1 bind MBF at the G2 phase of the cell cycle to restrict MBF transcriptional activity to the G1 phase. The genes encoding these co-repressors are themselves targets of MBF, so MBF negatively regulates its own activity after S phase.^22^^,^^23^^,^^24^^,^^25^^,^^26^^,^^27^

In *S. cerevisiae*, Whi5 is a transcriptional repressor that restricts SBF-dependent transcription to late G1 phase.^28^^,^^29^ A Whi5 paralog, Whi7/Slr3, has a role in the G1/S transition in response to stress^30^^,^^31^^,^^32^ and, together with Whi5 and other factors, regulates the transition to quiescence.^33^^,^^34^^,^^35^ Whi5 and Whi7 are functionally analogous to the mammalian tumor suppressor Retinoblastoma, an E2F repressor that cooperates with the RB-like protein p130 to repress transcription in G0. Understanding how gene expression is repressed during the cell cycle and in quiescence may provide valuable insight into cancer development and potential therapeutic strategies.^36^ Studies in model organisms such as yeast have helped to elucidate the underlying molecular mechanisms.

Based on sequence analysis, a candidate MBF repressor, orthologue of budding yeast Whi5, has been identified in fission yeast (http://www.pombase.org). In this work, we demonstrate that fission yeast Whi5 is a transcriptional repressor of the MBF complex in *S. pombe*. We found that, contrary to Nrm1, which down-regulates S-phase genes under nutrient rich conditions, fission yeast Whi5 plays a role in quiescent cells, downregulating the wave of early meiotic genes involved in premeiotic S-phase and recombination. Our work suggests that this function may be performed through the recruitment of histone deacetylase complex Clr6-I, pointing to a conserved role of this complex in the transcriptional repression of a subset of genes during quiescence and differentiation.

## Results

### Sequence homology suggests a role for fission yeast Whi5 as a co-repressor of MBF

Fission yeast cells have a cryptic size control in G1/S when cultured in a nitrogen-rich medium (YES or Minimal Medium with NH_4_Cl, MM). However, this control is activated, and the G1 phase is prolonged in cells growing in a nitrogen-poor medium (such as minimal medium with phenylalanine, MMPhe) or entering quiescence (minimal medium lacking nitrogen, MM-N). While most studies have examined the role of MBF in nitrogen-rich conditions, its function in nitrogen-poor conditions and in quiescent cells is less well understood. Previous work in our laboratory has demonstrated that, in MMPhe, inhibition of CDK activity in G1 is required for proper activation of MBF-dependent transcription at the end of G1.^37^^,^^38^ Just as important as generating adequate induction of MBF-dependent genes at the G1/S transition is their repression during G1 and quiescence. Other organisms, such as mammals, plants, or budding yeast, possess specific transcriptional inhibitors, Retinoblastoma in mammalian cells and plants, and Whi5 in *S. cerevisiae*, that play an important role in the entry and maintenance of quiescence.^28^^,^^29^^,^^33^^,^^39^^,^^40^

**Figure 1:**
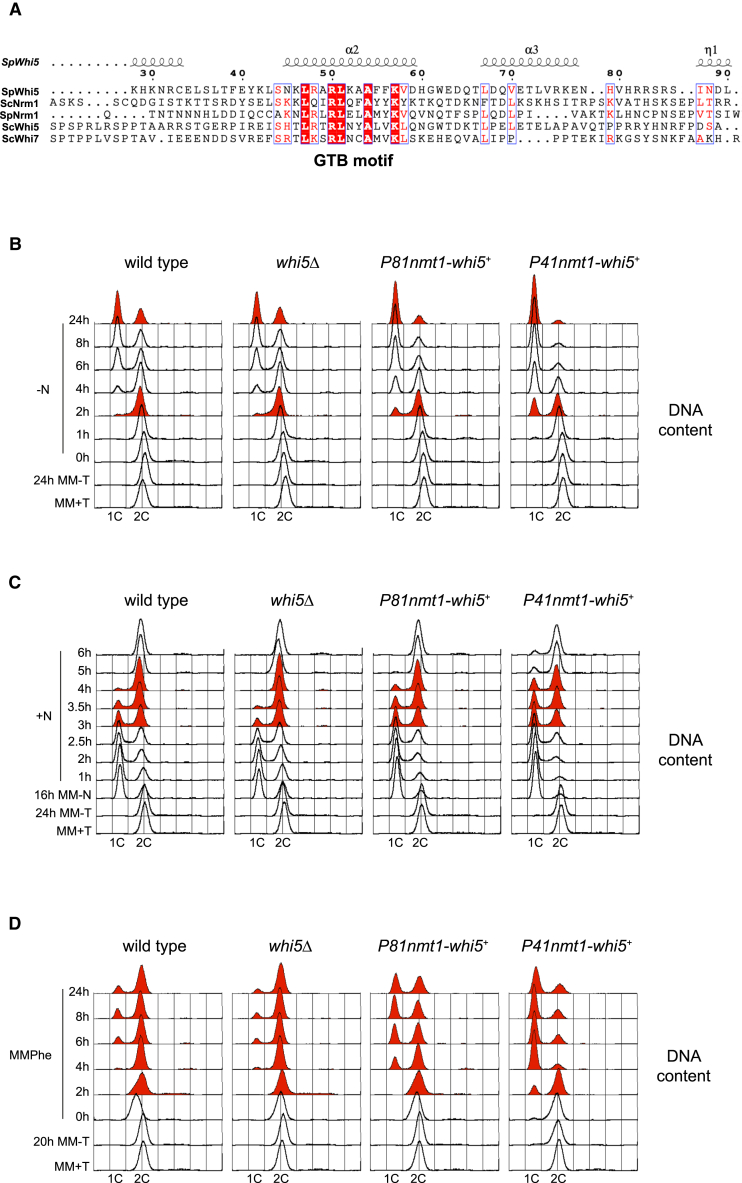
Whi5 levels regulate the entry and exit from quiescence by acting as a corepressor of MBF (A) Sequence alignment of *S. cerevisiae* Whi5, Whi7, and Nrm1, and *S. pombe* Whi5 and Nrm1, generated by Clustal Omega^43^ and represented with ESPript3.^44^ The GTB motif, which corresponds to an alpha-helix in the structure of the SpWhi5 protein, is highlighted. The structure of SpWhi5 predicted by Alphafold is shown over the sequence alignment.^45^ (B) FACS profiles show the DNA content of wild-type, *whi5Δ*, *P81nmt1-whi5*^*+*^, and *P41nmt1-whi5*^*+*^ cells entering quiescence. Cells were grown in MM with thiamine, washed twice with MM, and incubated in the same medium for 24 h to induce *whi5*^*+*^ expression from the *nmt1* promoter. Cells were then transferred to MM-N to induce entry into quiescence. (C) FACS analysis shows the DNA content of wild-type, *whi5Δ*, *P81nmt1-whi5*^*+*^, and *P41nmt1-whi5*^*+*^ cells upon quiescence exit. Expression of *whi5*^*+*^ from the *nmt1* promoter was induced as in (B). Cells were then transferred to MM-N for 16 h to induce entry into quiescence and then released by addition of NH_4_Cl. (D) Flow cytometry analysis shows the DNA content of wild-type, *whi5Δ*, *P81nmt1-whi5*^*+*^, and *P41nmt1-whi5*^*+*^ cells growing in minimal medium with phenylalanine as a nitrogen source (MMPhe). Cells were grown in MM without thiamine for 20 h at 32 °C to induce *whi5*^*+*^ expression from the *nmt1* promoter, and then shifted to MMPhe at the same temperature. Samples were taken at the indicated time points. See also Figures S1–S3.

*S. pombe* has a homologous protein to *S. cerevisiae* Whi5, named Whi5/Mug54 (referred to as Whi5 hereafter), described in Pombase as an SBF complex transcription corepressor. Like other SBF/MBF inhibitors in yeast and other organisms, Whi5 contains a G1/S transcription factor binding (GTB) motif that is supposed to interact with G1/S transcription factors^41^^,^^42^ (Figure 1A). In addition, *whi5*^*+*^ is termed *mug54*^*+*^ -*m*eiotically *u*pregulated *g*ene *54-* as it is induced during meiosis, although its deletion does not cause any apparent defect in sporulation.^1^^,^^46^

We deleted *whi5*^*+*^ and observed no apparent phenotype when cells were grown in YES or MM, indicating that *whi5*^*+*^ is probably dispensable in nitrogen-rich media. We then examined growth in cells entering quiescence by removing the nitrogen source from the medium. Under these conditions, *whi5Δ* cells showed no significant differences in the G1 population (Figure 1B). In contrast, *whi5*^*+*^*-*overexpressing cells arrested in G1 earlier than the wild type (Figure 1B; see *P81nmt1-whi5*^*+*^ and *P41nmt1-whi5*^*+*^ at 2 h in MM-N), and we observed that after 24 h of overexpression there were almost no cells with a 2C DNA content. This phenotype was dose dependent as it was more severe as we increased the level of overexpression (compare the moderate *P41nmt1* promoter with the milder *P81nmt1* promoter). This result is consistent with the idea that Whi5 may play a role as a transcriptional repressor at the G1/S transition.

Next, to confirm these results, we analyzed the behavior of *whi5Δ* and *whi5*^*+*^*-*overexpressing cells at exit from quiescence. Consistent with a role for *whi5*^*+*^ in promoting G1 arrest and quiescence, we observed that *whi5*^*+*^*-*deleted cells completed S-phase earlier than the wild type (Figure 1C, 3-4 h). In contrast, cells with high levels of *whi5*^*+*^ showed a delay in G1 exit compared to wild type (Figure 1C, see *P81nmt1-whi5*^*+*^ and *P41nmt1-whi5*^*+*^ at 3–6 h). Again, *P41nmt1-whi5*^*+*^ cells, expressing higher levels of *whi5*^*+*^, showed a more severe phenotype, and 4–6 h after exiting arrest, a percentage of cells still exhibited 1C DNA content.

To further explore this phenotype, we examined the growth of these cells in minimal medium containing phenylalanine as a nitrogen-poor source. Flow cytometry analysis revealed that the *whi5Δ* mutant showed a slightly reduced percentage of cells with a 1C DNA content, while *P81nmt1-whi5*^*+*^ and *P41nmt1-whi5*^*+*^ mutants exhibited a higher percentage of cells in G1 (Figure 1D). Consistent with this phenotype, in other media with a nitrogen-poor source, such as minimal medium with isoleucine, the *whi5Δ* mutant showed a slightly reduced percentage of cells with a 1C DNA content (Figure S1A). Furthermore, we observed that in MMPhe, while *whi5Δ* cells reduced in size as did wild-type cells, the size of *whi5*^*+*^-overexpressing cells increased (Figure S1B). Previous work in our laboratory has shown that proper activation of MBF in MMPhe is necessary to prevent replicative stress and DNA damage, as cells lacking the MBF activator *rep2*^*+*^ do not reduce cell size as the wild-type cells but instead elongate, as a result of activation of DNA integrity checkpoints.^37^^,^^38^ This result suggests that the overexpression of *whi5*^*+*^, a putative repressor of MBF, may be mimicking *rep2Δ* cells, pointing to a reduction in MBF-dependent gene expression as a likely cause of replicative stress and checkpoint activation in *whi5*^*+*^*-*overexpressing cells. Accordingly, we observed an increased number of Rad52-YFP foci in *whi5*^*+*^-overexpressing cells compared to the wild type in MMPhe (Figure S1C). Taken together, these findings suggest that Whi5 plays a non-essential role in establishing quiescence under nitrogen starvation, as well as in modulating the G1/S transition in nitrogen-poor medium.

Next, we analyzed the genetic interactions of *whi5Δ* with components of the MBF complex. Consistent with published screens, deletion of *whi5*^*+*^ increases the fitness of *res1Δ*^47^^,^^48^ (Figure S2A), and we observed this phenotype in both nitrogen-rich and nitrogen-poor media (YES, MM, and MMPhe; Figure S2A). In addition, in the case of the MBF activator *rep2Δ*, whose growth defect was less severe, we also observed a partial suppression by *whi5Δ* in the same media (Figure S2A). Furthermore, we found that the elongation phenotype of both *res1Δ* and *rep2Δ* in MMPhe is largely suppressed by *whi5*^*+*^ deletion (Figures S2B and S2C), suggesting reduced replication stress and DNA damage in the double mutants *res1Δ whi5Δ* and *rep2Δ whi5Δ* compared with *res1Δ* and *rep2Δ*, respectively, probably as a result of increased MBF-dependent gene expression. Consistently, *whi5Δ* partially suppressed the elongation phenotype of the temperature-sensitive *cdc10-129* mutant at a semi-restrictive temperature in MMPhe (Figure S2D). By contrast, deletion of *whi5*^*+*^ did not suppress the phenotype of the *res2Δ* mutant in MMPhe (Figure S2E). Conversely, overexpression of *whi5*^*+*^ exacerbated the growth and elongation defects of the *res1Δ*, *rep2Δ,* and *cdc10-129* mutants even in MM, but not that of the *res2Δ* mutant (Figures S3A, S3B, S3C, and S3D). In the case of *cdc10-129* and *res2Δ*, these effects were more pronounced in MMPhe (Figure S3E). To further explore these phenotypes, we analyzed the formation of DNA repair Rad52 foci in *rep2Δ* and *rep2Δ whi5Δ* in MMPhe. Consistent with our published results, *rep2Δ* showed increased Rad52-YFP foci formation after 20 h of growth in MMPhe^38^ (Figure S2F). Interestingly, in the *rep2Δ whi5Δ* double mutant, the number of Rad52 foci was almost reduced to wild-type levels (Figure S2F). Taken together, these results support a role for Whi5 as a transcriptional repressor of MBF in nitrogen-poor media where the G1/S size control is activated.

### Whi5 downregulates the expression of a subset of MBF-dependent genes in quiescent cells

**Figure 2:**
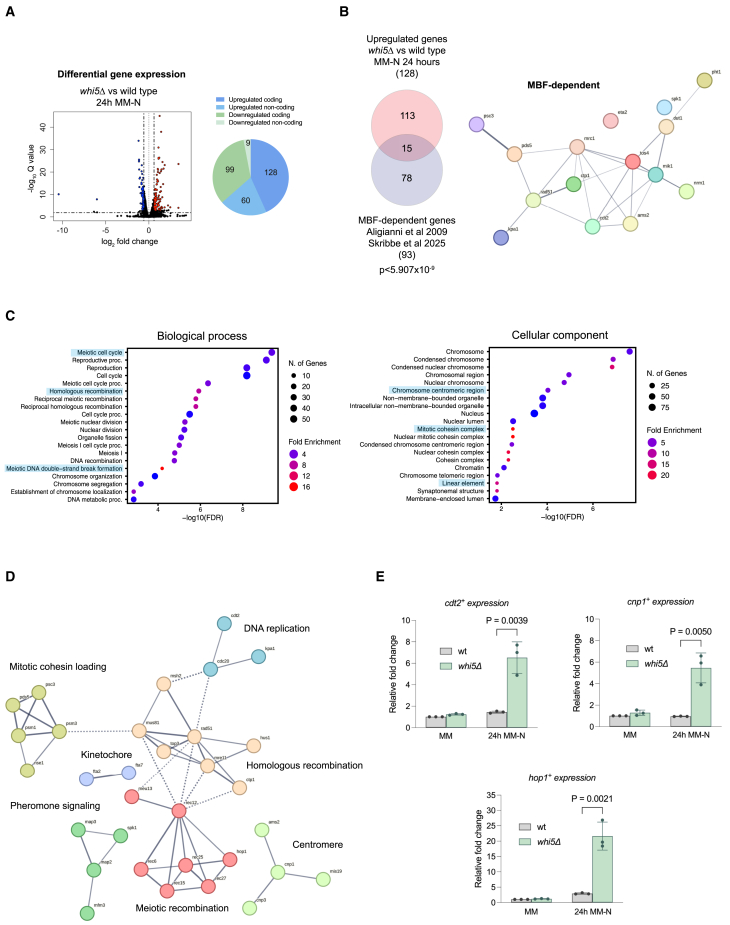
Upregulation of MBF-dependent early meiotic genes in *whi5Δ* cells under nitrogen starvation (A) Volcano plot shows differentially expressed genes in *whi5Δ* versus wild-type cells at 24 h in MM-N. Genes significantly upregulated (adjusted *p* - value<0.001, fold change>2^0.^^585^) and significantly downregulated (adjusted *p* - value<0.001, fold change<2^−0.585^) are represented by red and blue dots, respectively, while the remaining genes are depicted as black dots. The pie chart shows the number of protein-coding and non-coding genes among up- and downregulated genes. (B) Venn diagrams show the overlap of overexpressed codifying genes in *whi5Δ* compared to the wild-type cells at 24 h in MM-N, and MBF-dependent genes.^22^^,^^49^*p* - values were calculated using a hypergeometric test. Drawing depicting MBF-dependent genes upregulated in *whi5Δ* made with the webtool String. (C) Gene ontology (GO) analysis of genes upregulated in *whi5Δ* at 24 h in MM-N. Enriched GO terms for Biological processes and Cellular components are shown. Analyses were done with the Shinygo website (FDR<0.05), and representative GO terms are highlighted. (D) Visualization of a subset of upregulated genes from the enriched categories highlighted in C using the String website. Insertion of the antibiotic resistance cassette at the *whi5*^*+*^ locus increases the expression of the *clr3*^*+*^ gene, which is located next to *whi5*^*+*^. Therefore, *clr3*^*+*^ was excluded from this and subsequent analyses. (E) Validation of RNAseq results. The *cdt2*^*+*^, *cnp1*^*+*^, and *hop1*^*+*^ genes were selected for validation by RT-qPCR. Bars represent the fold change in expression relative to wild-type cells in MM. Data represent mean values from three biological replicates, and error bars represent standard deviations. *p*-values were calculated using a Student’s *t* test (unpaired, two-tailed). See also Figure S4 and Tables S1 and S2.

To understand the function of Whi5 during quiescence entry, we analyzed by RNAseq the transcriptome of wild-type and *whi5Δ* cells cultured in MM and for 10 or 24 h in MM-N. In total, 19 genes were differentially expressed in *whi5Δ* versus wild-type cells in MM, 149 genes at 10 h in MM-N, and 296 genes at 24 h in MM-N (Figure 2A; Table S1). According to the predicted function of Whi5 as a transcriptional repressor and to the observed phenotype in quiescence (Figures 1B and 1C), the main differences were found in the group of genes upregulated at 10 and 24 h in MM-N (124 and 188 upregulated genes, respectively; Figure 2A; Table S1). Interestingly, among the upregulated genes we found MBF-dependent genes, as well as genes potentially regulated by the MBF complex^22^^,^^49^^,^^50^ (Figure 2B). We performed GO enrichment analysis to find overrepresented categories in the list of genes upregulated in *whi5Δ* at 24 h in MM-N (Figure 2C; Table S2). We observed that, in addition to Cdc10 targets that function in both mitosis and meiosis, such as the ubiquitin ligase *cdt2*^*+*^, the chromatin binding FHA domain protein *tos4*^*+*^, genes involved in cohesion (*psc3*^*+*^, *pds5*^*+*^) and centromeric function (*cnp1*^*+*^), there was an overrepresentation of early meiotic genes involved in functions such as meiotic recombination, double-strand break formation, and linear element formation (*hop1*^*+*^, *rec12*^*+*^, *meu13*^*+*^, *rec27*^*+*^ …) (Figures 2C and 2D; Table S2). To confirm these results, we validated some of the upregulated genes by qPCR (Figure 2E).

During meiosis, a specific MBF complex, composed of Cdc10, Res2, and Rep1, is required for the expression of genes involved in premeiotic S-phase and recombination.^15^^,^^51^^,^^52^ Our results suggest that, under nitrogen starvation, Whi5 may be required to maintain early meiotic gene expression at low levels until pheromone signaling induces entry into meiosis. However, in the absence of cells of the opposite mating type (i.e., no pheromone signaling), but with nitrogen availability restored, cells would resume proliferation. Interestingly, ectopic expression of meiotic genes in vegetative cells is toxic and, in *S. pombe*, overexpression of *spo11*^*+*^ or *rec8*^*+*^ in proliferating cells can lead to centromere dismantlement and kinetochore loss.^53^ To prevent this, cells have evolved mechanisms to selectively remove meiotic transcripts in vegetative cells, such as the YTH-family RNA-binding protein Mmi1 and the exosome.^54^ Given the importance of meiotic transcript elimination in proliferative cells and since these genes are overexpressed in *whi5Δ* during quiescence, we analyzed their levels in a quiescent exit experiment. We measured *rec8*^*+*^ and *rec12*^*+*^ expression by qPCR upon exit from quiescence in the wild type and in *whi5*^*+*^-deleted cells, and, despite the increased expression of these genes in the *whi5Δ* mutant in MM-N, we observed a rapid disappearance of these transcripts during the first hour after release (Figures S4A, S4B, and S4E). These results suggest that Whi5 is not required to repress meiotic gene expression upon exit from quiescence.

In the RNAseq analysis, we did not find a significant increase in the expression of MBF-dependent replication genes *cdc18*^*+*^ and *cdc22*^*+*^ after 24 h under nitrogen starvation. However, some MBF targets with a function in S-phase were upregulated (Figure 2B; Tables S1 and S2). As we observed a slight acceleration in completion of DNA replication in *whi5Δ* upon exit from quiescence (Figures 1C and S4E), we speculate that this could be due to an early onset of DNA replication or an accelerated progression of replication due to the overexpression of MBF-dependent S-phase genes in *whi5Δ* cells upon exit from quiescence. To explore this possibility, we analyzed by qPCR the expression of two S-phase genes, *cdc18*^*+*^ and *cdt2*^*+*^. Cdc18 is a critical target of MBF that is required to initiate DNA replication, and whose overexpression can overcome the G1 arrest of a *cdc10ts* mutant.^55^^,^^56^ Cdt2 is a substrate recognition adaptor of the CRL4 E3 ubiquitin ligase complex, which targets the degradation of numerous proteins in S-phase, such as the ribonucleotide reductase inhibitor Spd1. Interestingly, Spd1 degradation can be induced by the overexpression of *cdt2*^*+*^.^57^^,^^58^ Expression of *cdc18*^*+*^ and *cdt2*^*+*^ genes was analyzed in MM, after 16 h in MM-N, and every 30 min after the release from the G1-block for 4 h (Figures S4C, S4D, and S4E). In the case of *cdc18*^*+*^, we observed that in the wild type, *cdc18*^*+*^ expression peaks at 120 min, just before S-phase (Figures S4C and S4E). In the *whi5Δ* mutant, *cdc18*^*+*^ expression is only slightly higher in quiescent cells (16h in MM-N) compared to the wild type and peaks earlier, at 90 min after the release from the G1-block (Figures S4C and S4E). Regarding *cdt2*^*+*^, we observed that in the *whi5Δ* mutant, *cdt2*^*+*^ expression is very high in MM-N compared to the wild type and is maintained at this level during the G1 phase, and until S phase, after which *cdt2*^*+*^ expression decreases in *whi5Δ* with similar kinetics to the wild type (Figures S4D and S4E). In summary, as shown in the RNAseq analysis in quiescent cells, *cdc18*^*+*^ does not appear to be significantly upregulated in *whi5Δ* upon exit from quiescence, although it peaks earlier. By contrast, *cdt2*^*+*^ expression is very high in G1 cells upon exit from quiescence and may contribute to the altered kinetics observed in *whi5Δ* cells.

### Whi5 localizes to the nucleus and cooperates with Nrm1 in repressing MBF-dependent transcription

**Figure 3:**
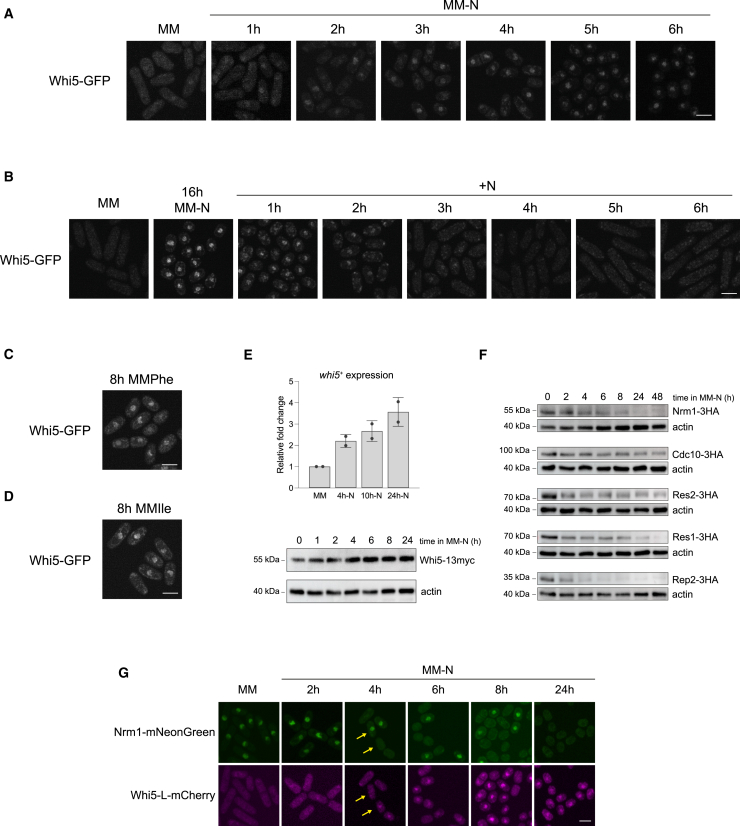
Whi5 localizes to the nucleus under nitrogen starvation and in nitrogen-poor media (A) Localization of Whi5-GFP in cells entering quiescence. Cells were cultured in MM at 25 °C until exponential growth, washed twice in MM-N, and transferred to MM-N at 25 °C for 6 h. Fluorescence confocal images were taken at the indicated time-points. Maximum intensity projections from stacks of 8 images are shown. Scale bar: 5 μm (B) Localization of Whi5-GFP during quiescence exit. Fluorescent images of Whi5-GFP in MM, after 16 h in MM-N, and at 1, 2, 3, 4, 5, and 6 h following the release from the G1 block by the addition of NH_4_Cl. The nitrogen starvation block was done at 25 °C, and the block release was at 32 °C. Scale bar: 5 μm (C) Localization of Whi5-GFP after 8 h of growth in MMPhe at 25°C. Scale bar: 5 μm (D) Localization of Whi5-GFP after 8 h of growth in MMIle at 25°C. Scale bar: 5 μm (E) Analysis of *whi5*^*+*^ expression by qPCR (upper panel) and Whi5 protein levels by Western blot (lower panel). Samples were collected at the indicated timepoints for RNA or protein extraction. Bars represent fold change in expression relative to MM. The mean and standard deviations from two biological replicates are shown. Protein extracts were analyzed by SDS-PAGE and Western blot. Whi5-13myc was detected with anti-myc antibodies. Actin was used as a loading control. Western blot was repeated twice, and a representative experiment is shown. (F) Western blot analysis of Nrm1-3HA, Cdc10-3HA, Res2-3HA, Res1-3HA, and Rep2-3HA during entry into quiescence. Samples were collected from cells growing in MM, and in MM-N every 2 h for the first 8 h, and then at 24 and 48 h. Protein extracts were analyzed by SDS-PAGE, followed by western blotting and immunodetection using anti-HA antibodies. Actin was used as a loading control. A representative experiment is shown (*n* = 2). (G) Analysis of Whi5 and Nrm1 localization during nitrogen starvation. Fluorescence images of the Whi5-L-mCherry Nrm1-mNeonGreen strain growing in MM and at 2, 4, 6, 8, and 24 h of nitrogen starvation. Stacks of 8 images were obtained by confocal microscopy, and maximum intensity projections are shown. Scale bar: 5 μm. Yellow arrows highlight cells with Nrm1 or Whi5 nuclear localization. See also Figures S5–S8.

**Figure 4:**
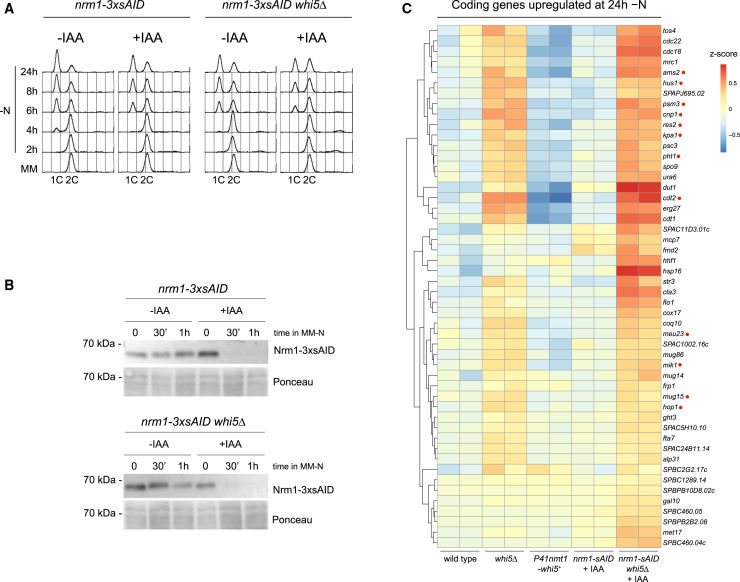
Nrm1 contributes to the downregulation of MBF-dependent genes in MM-N (A) FACS profiles show the DNA content of *nrm1-3xsAID* and *nrm1-3xsAID whi5Δ* cells entering quiescence in the presence or absence of 5-adamantyl-IAA, which induces the degradation of Nrm1. Cells were grown in MM to exponential growth and then transferred to MM-N to induce entry into quiescence. Samples were collected at the indicated time-points and DNA content was analyzed by flow cytometry. (B) Western blot analysis of *nrm1-3xsAID* and *nrm1-3xsAID whi5Δ* cells. Samples were collected from cells growing in MM and in MM-N for 30 and 60 min in the presence of 100 nM 5-adamantyl-IAA. As a control, samples from a culture without the drug were collected at the same time-points. Protein extracts were analyzed by SDS-PAGE followed by western blotting and immunodetection with anti-AID antibodies. Ponceau staining was used as a loading control. (C) Heatmap shows z-scores of the top 50 upregulated genes in the *whi5Δ nrm1-3xAID* vs. wild type at 24 h in MM-N (1.5 fold-change, adjpvalue<0.05), in the strains wild type, *whi5Δ*, *P41nmt1-whi5*^*+*^, *nrm1-3xAID*, and *whi5Δ nrm1-3xAID*. Genes from the GO term “meiotic cell cycle” (GO0051321) and genes induced in early meiosis^1^ are highlighted.

**Figure 5:**
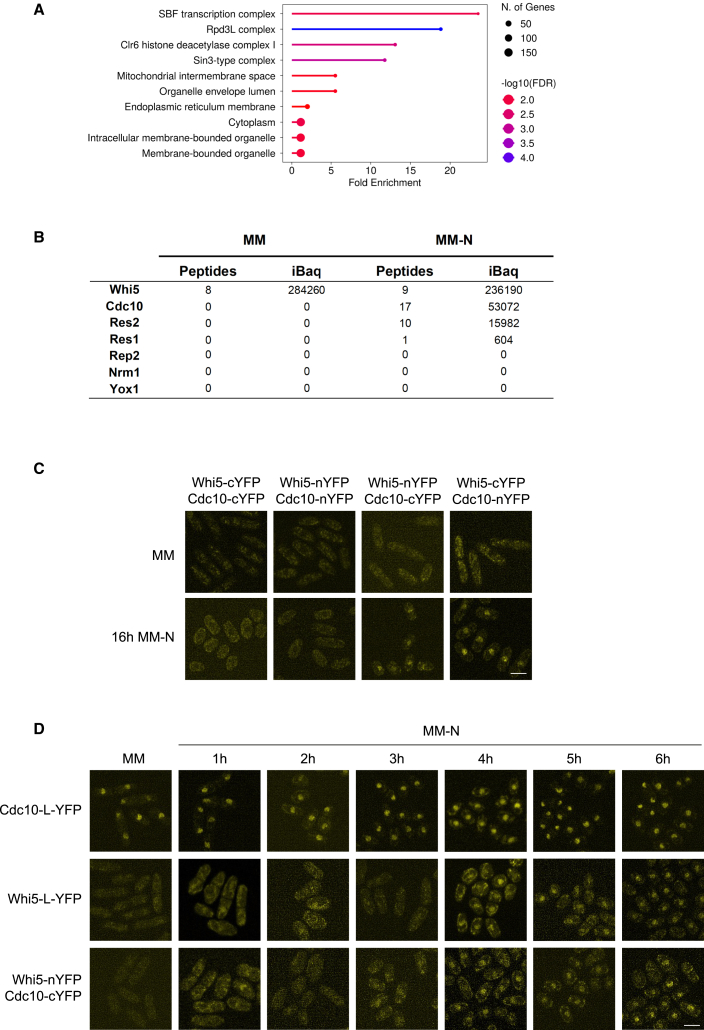
Whi5 interacts with the MBF complex during quiescence (A) Enrichment analysis of interactors of Whi5-GFP at 24 h in MM-N. The analysis shows the top ten enriched Cellular components (Shinygo web tool, FDR<0.05). (B) Summary table of the results obtained for the components of the MBF complex in the mass spectrometry analysis of Whi5-GFP interactors in MM and after 16 h in MM-N. The full dataset is provided in Table S3. (C) BiFC analysis of Cdc10 and Whi5 interaction in MM and after 16 h under nitrogen starvation. *whi5-nYFP cdc10-cYFP*, *whi5-cYFP cdc10-nYFP,* and control strains *whi5-nYFP cdc10-nYFP* and *whi5-cYFP cdc10-cYFP* were grown in MM and then for 16 h in MM-N. YFP fluorescence was analyzed by confocal microscopy. Maximum intensity projections of stacks of 8 images are shown. Scale bar: 5 μm (D) BiFC analysis of Whi5 and Cdc10 interaction at entry into quiescence. *whi5-L-YFP*, *cdc10-L-YFP,* and *whi5-nYFP cdc10-cYFP* strains were grown in MM and transferred to MM-N for 6 h. Fluorescence images were taken at the indicated time points. Maximum intensity projections of 8 slices with a Z-step of 0.2 μm are shown. Scale bar: 5 μm See also Figures S9 and S10 and Table S3.

**Figure 6:**
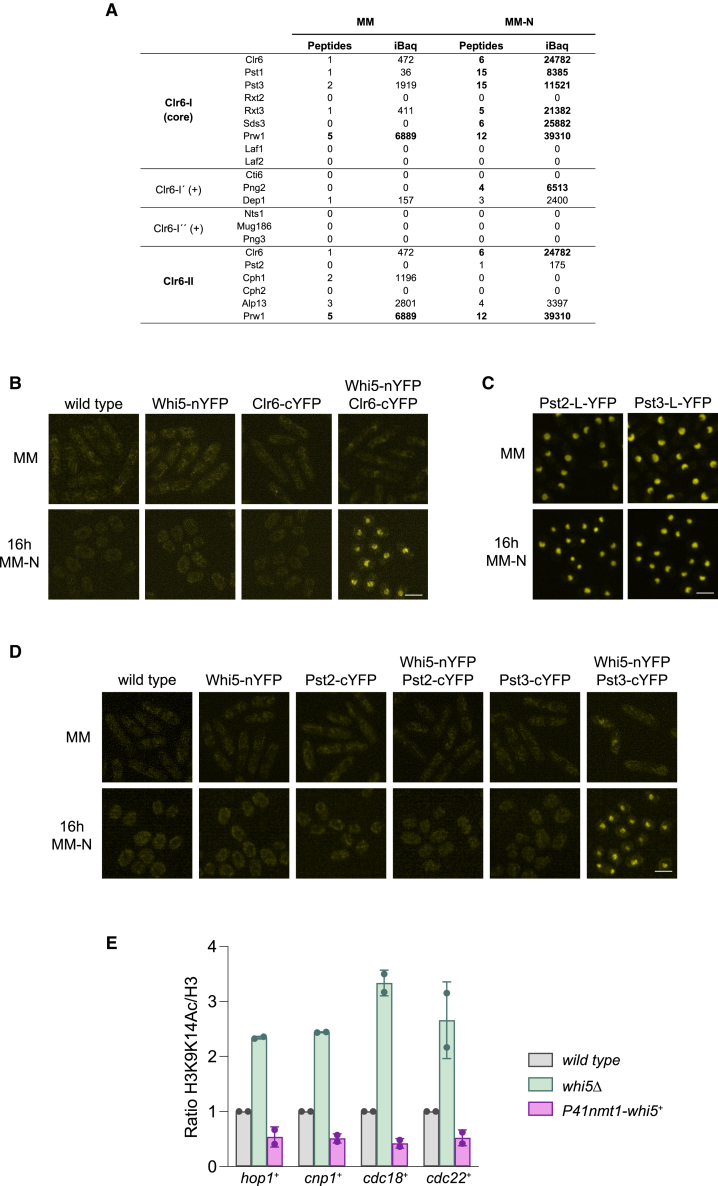
Interaction of Whi5 with components of the histone deacetylase complex Clr6-I (A) Mass spectrometry analysis of the Whi5-GFP interactome. Summary table of the components of Clr6-I and Clr6-II complexes identified. Whi5-GFP was immunoprecipitated from cells growing in MM and after 16 h in MM-N. Components of the Clr6-I complex with iBAQ>4000 are highlighted in bold. Full dataset is provided in Table S3. (B) Analysis of Whi5 and Clr6 interaction by BiFC. The double-tagged *whi5-nYFP clr6-cYFP* strain and control strains (wild-type, *whi5-nYFP,* and *clr6-cYFP*) were grown in MM, and then transferred to MM-N and incubated for 16 h at 25 °C. Stacks of 8 confocal fluorescence images were taken, and maximum intensity projections are shown. Scale bar: 5 μm (C) Localization of Pst2-L-YFP and Pst3-L-YFP in MM and MM-N (16 h). Scale bar: 5 μm (D) BiFC analysis of interactions between Whi5 and Pst2 or Pst3. Strains wild type, *whi5-nYFP, pst2-cYFP, whi5-nYFP pst2-cYFP, pst3-cYFP,* and *whi5-nYFP pst3-cYFP* were grown in MM (upper panels), and in MM-N for 16 h at 25 °C (bottom panels). Scale bar: 5 μm (E) Analysis of H3 acetylation of the promoter regions of *hop1*^*+*^, *cnp1*^*+*^, *cdc18*^*+*^, and *cdc22*^*+*^ genes by ChIP in the wild type, *whi5Δ,* and *P41nmt1-whi5*^*+*^ strains. Expression from the *nmt1* promoter was induced for 24 h in MM without thiamine, and then, cultures were shifted to MM-N to induce quiescence entry. Samples were collected after 16 h of nitrogen starvation and processed for ChIP. Chromatin was immunoprecipitated with antibodies against acetylated lysines K9 and K14 on histone H3, and with antibodies against total histone H3. The H3K9K14-Ac/H3 ratio and the fold change relative to the wild-type strain were calculated. Data represent the means and standard deviations of two biological replicates. See also Figures S9 and S10 and Table S3.

In *S. cerevisiae,* Whi5 is known to shuttle between the nucleus and the cytoplasm, showing nuclear accumulation in G1, from mitotic exit to the G1/S transition.^28^ Increased nuclear localization of Whi5 has also been observed in response to nutrient depletion and other environmental stresses.^59^^,^^60^ In the case of ScWhi7, a paralog of Whi5, although it accumulates in the nucleus in G1, never becomes completely nuclear, but remains distributed between the nucleus and the cytoplasm.^31^ Indeed, Whi7 has been shown to play a role in G1 bound to the ER membrane.^32^ To analyze the cellular localization of Whi5 in *S. pombe*, we constructed a functional version of Whi5 C-terminally tagged with GFP expressed under its own promoter (Figure S5) and analyzed its distribution in cells upon entry into and exit from quiescence (Figures 3A and 3B). We found that Whi5 localized throughout the cell in exponentially growing cells in nitrogen-rich medium (MM) (Figures 3A and 3B, first panels). Interestingly, Whi5 accumulated rapidly in the nucleus when cells were transferred to MM-N (Figure 3A). Nuclear localization began within the first few hours after the shift to MM-N and increased progressively over time (Figures 3A, 1-6 h; 3B, 16 h; S6). Furthermore, as observed for Whi7 in *S. cerevisiae*, Whi5 distribution in MM-N was not exclusively nuclear, as a fraction of the protein remained in the cytoplasm (Figure S6A). After release from quiescence, we observed a rapid reduction in the nuclear accumulation of Whi5-GFP (Figures 3B and S6, 1-6 h). Similarly, we analyzed the localization of Whi5-GFP in nitrogen-poor media, MMPhe and MMIle, and observed that the concentration of Whi5-GFP increased in the nucleus (Figures 3C and 3D; S7).

Next, we analyzed the mRNA and protein levels of Whi5 by qPCR and western blot, respectively, and observed increased levels in MM-N compared to MM (Figures 3E and S8). To characterise the MBF complex in quiescence, we examined the protein levels of each of its components during nitrogen starvation (Figures 3F and S8). According to previously published data, the protein levels of Cdc10 and Res2 are maintained under nitrogen starvation.^15^^,^^17^ In contrast, Res1, which is absent during meiosis, reduces its levels and is almost undetectable at later time points in quiescent cells (48 h in nitrogen-depleted medium, Figure 3F). The Rep2 coactivator, whose expression decreases under nitrogen starvation,^20^ disappears during the first hours in MM-N. The MBF repressor Nrm1 also disappears shortly after the mitotic divisions that precede G1 arrest.^61^ In *S. cerevisiae*, Whi5 and Nrm1 have been shown to bind to Swi6 through the GTB motif.^42^ Similarly, we propose that Whi5 may bind to Cdc10 through its GTB motif in nitrogen-depleted medium, when the Nrm1 repressor is no longer produced. To test this hypothesis, we analyzed a Whi5-L-mCherry Nrm1-mNeonGreen double-tagged strain in a time-course in MM-N (Figure 3G). We observed a time-dependent decrease in Nrm1-mNeonGreen fluorescence and an increase in Whi5-L-mCherry nuclear signal. In addition, we observed that the increase in Whi5-L-mCherry levels in MM-N correlated with the decrease in Nrm1-mNeonGreen levels (Figure 3G; see yellow arrows). These results suggest that, in response to the shift from a nitrogen-rich to a nitrogen-depleted medium – leading to a decrease in G2 cells and an increase in G1 cells – Whi5 becomes the main repressor of MBF in G1 cells under nitrogen starvation. We observed that Nrm1 remains present in MM-N during the mitotic divisions preceding G1 arrest (Figures 3F and 3G) and thus Nrm1, together with Whi5, may contribute to the silencing of MBF-dependent genes upon entry into quiescence. To further explore this possibility, since *nrm1Δ* cells exhibit high levels of genomic instability, we constructed a degron allele of *nrm1*^*+*^ to induce Nrm1 degradation after the shift from MM to MM-N (Figures 4A and 4B).^62^ As shown in Figure 4A, degradation of Nrm1 following degron induction induces a delay in G1 arrest in MM-N, and this phenotype was more severe in the absence of both Nrm1 and Whi5 (Figure 4A; compare *nrm1-3xsAID whi5Δ* with and without auxin at 24 h). These results indicate that both repressors contribute to the correct entry into quiescence.

To get insight into the shared role of Nrm1 and Whi5 in the repression of MBF-dependent transcription at entry into quiescence, we performed an RNAseq experiment to analyze the transcriptome of the *nrm1-3xsAID* and *nrm1-3xsAID whi5Δ* mutants after 24 h in MM-N in the presence of auxin. As a control, we analyzed the transcriptome of *whi5*^*+*^-deleted and *whi5*^*+*^-overexpressing cells (Figure 4C). We observed increased expression of MBF-dependent genes in the double mutant *nrm1-3xsAID whi5Δ* compared to single mutants *nrm1-3xsAID* and *whi5Δ*, suggesting that both repressors have a synergistic effect under nitrogen starvation (Figure 4C).

### Whi5 physically interacts with the MBF complex in nitrogen-depleted medium

To obtain *in vivo* evidence of Whi5 interaction with the MBF complex, we performed a mass spectrometry analysis of proteins co-purified with Whi5 in MM and after 16 h in MM-N (Figures 5A and S9A; Table S3). We found that, specifically under nitrogen starvation, as expected from our localization and functional analyses, Whi5 interacted with two components of the MBF complex, Cdc10 and Res2, whereas no significant interaction was observed with Res1 (Figures 5B and S9A). As shown in Figure 3F, Res1, which is absent in meiosis, greatly diminishes under nitrogen starvation, and it is almost undetectable after 24–48 h in MM-N. To determine MBF composition and, specifically, if Res1 was present in the MBF complex at the time we analyzed Whi5 interactome (16 h in MM-N), and to confirm the presence of Whi5 in the complex, we immunoprecipitated Cdc10-L-YFP at this time point. As expected, Whi5 was among the interactors of Cdc10 in MM-N (Tables 1 and S4). In addition, we found that both Res1 and Res2 co-immunoprecipitated with Cdc10, with Res2 being more abundant (Tables 1 and S4; S9B). These results suggest that Whi5 is a specific repressor of the Cdc10-Res2 complexes in quiescent cells (Figure 5B; Tables 1, S3 and S4).

**Table 1:** List of proteins of the Clr6 and the MBF complexes detected in the mass spectrometry analysis of Cdc10-L-YFP in a wild type and in *whi5Δ* after 16 h in MM-N

		*cdc10-L-YFP*		*cdc10-L-YFP whi5Δ*	
		Peptides	iBaq	Peptides	iBaq
MBF	Cdc10	29	159400	30	113430
	Res2	23	103460	26	79710
	Res1	15	59469	6	16873
	Whi5	4	21228	0	0
Clr6-I	Clr6	8	15955	0	0
	Pst1	10	3747	0	0
	Pst3	11	6195	0	0
	Rxt3	2	11994	0	0
	Sds3	4	21973	0	0
	Prw1	7	16630	1	284
	Png2	3	2841	0	0

Full dataset is given in Table S4.

The high levels of protease activity in nitrogen-starved cells precluded analysis of the interaction of Whi5 with the MBF complex by Co-ImmunoPrecipitation (Co-IP) followed by western blot. Instead, we confirmed the interaction of Whi5 with the MBF complex *in vivo* by Bifunctional Fluorescence Complementation (BiFC) assay.^63^^,^^64^^,^^65^ For this assay, the n-YFP and c-YFP fragments of the yellow fluorescent protein were fused to Cdc10 and Whi5 at the C-terminus, and the tagged alleles were combined by genetic crosses to obtain the corresponding double-tagged strains. To test whether protein tagging affects the functionality of Cdc10, which is an essential protein, we grew the strains at different temperatures and found no difference in growth between the tagged alleles and the wild type (Figure S10A). Next, we used the generated strains to analyze YFP fluorescence in MM and after 16 h in MM-N. Confirming our mass spectrometry results, only in MM-N did we detect interaction between Cdc10 and Whi5 (Figure 5C; see YFP signal in *whi5-cYFP cdc10-nYFP* and *whi5-nYFP cdc10-cYFP* in MM-N). In addition, to accurately determine the timing of the Whi5-Cdc10 interaction after the shift to MM-N, we perform a time-course experiment including strains *whi5-L-YFP*, *cdc10-L-YFP,* and *whi5-nYFP cdc10-cYFP*. In agreement with published results, a stable signal of Cdc10-L-YFP was detected in the nucleus both in MM and MM-N^15^ (Figure 5D). As demonstrated above, Whi5-L-YFP was detected in the nucleus after the shift to MM-N (at 3–4 h in this time-course), and the Whi5-Cdc10 interaction was detected approximately at the same time, suggesting that as soon as Whi5 enters the nucleus, it interacts with the MBF complex (Figure 5D).

### Transcriptional repression by Whi5 may be mediated by the recruitment of the histone deacetylase complex Clr6-I

Enrichment analysis of proteins detected in the interactomes of Whi5 and Cdc10 revealed that both proteins interact with components of the Clr6-I complex in MM-N (Figures 6A and S9; Tables 1, S3 and S4). Clr6 is a histone deacetylase that forms part of two main complexes, Clr6-I and Clr6-II.^66^^,^^67^ While the Clr6-I complex deacetylates histones at promoter regions, the Clr6-II complex targets coding regions, preventing cryptic transcription.^66^^,^^68^ Interestingly, Whi5 in *S. cerevisiae* and RB in human cells function as transcriptional repressors in part through the recruitment to chromatin of HDACs, Rpd3 in *S. cerevisiae* and HDAC1 in human cells.^69^^,^^70^^,^^71^ This result pointed to a conserved role of fission yeast Whi5 in transcriptional repression through deacetylation of histones at gene promoters. This prompted us to further investigate these results, first, confirming the interaction of Whi5 with Clr6 by BiFC (Figure 6B). In this case*,* Clr6 tagged with n-YFP was not functional and produced non-viable cells, as the *clr6*^*+*^ gene is essential.^72^ Thus, we performed the analysis with *whi5-nYFP clr6-cYFP*, *clr6-cYFP,* and *whi5-nYFP*, as these strains did not show growth defects at different temperatures (Figure S10B). As shown in Figure 6B, Whi5 interaction with Clr6 in MM-N was confirmed by BiFC. Furthermore, we detected a weak YFP nuclear fluorescence signal in nitrogen-rich medium (MM), according to the reduced level of interaction detected by mass spectrometry (Figures 6A and 6B; Table S3). Clr6 forms part of both Clr6-I and Clr6-II complexes. However, mass spectrometry analysis indicated that Whi5 mainly interacts with the Clr6-I complex. To confirm this specific interaction, we performed BiFC analysis using specific components of these complexes, Pst3 in the case of Clr6-I, and Pst2 in the case of Clr6-II. Both Pst2 and Pst3 are expressed in MM and MM-N and show nuclear localization (Figure 6C). After analyzing the functionality of BiFC strains (Figures S10C and S10D), we grew cells in MM and MM-N for 16 h. As shown in Figure 6D, Whi5 specifically interacted with Pst3 (Clr6-I complex) but not with Pst2 (Clr6-II complex) in quiescent cells. As with Clr6, the interaction between Whi5 and Pst3 was detected mainly in MM-N and only slightly in MM (Figure 6D). All these data suggest that in *S. pombe,* Whi5 may be repressing transcription through the recruitment of the Clr6-I complex to the promoter regions of specific genes. According to this, when we immunoprecipitated Cdc10-L-YFP at 16 h in MM-N in the *whi5Δ* mutant and analyzed the interactome by mass spectrometry, we found that the interaction of Cdc10-L-YFP with the Clr6-I complex was abolished in the absence of *whi5*^*+*^ (Tables 1 and S4). These results indicate that Whi5 is essential for the recruitment of HDACs by the MBF-complex.

**Figure 7:**
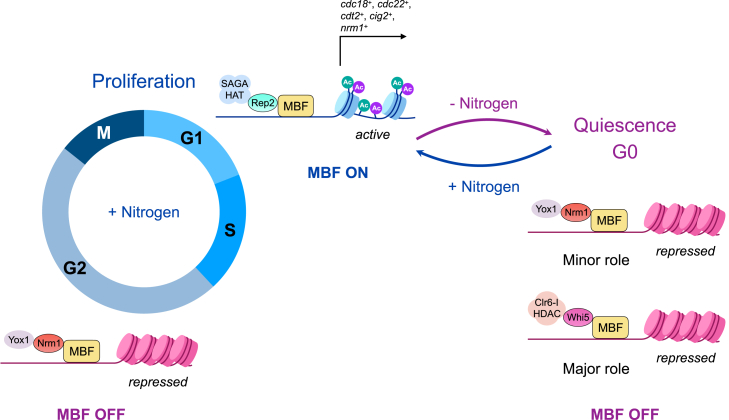
Model of regulation of MBF-dependent transcription during entry into and exit from quiescence During entry into quiescence (-Nitrogen), Whi5, and Nrm1 collaborate to repress the MBF complex, with Whi5 playing a major role. Whi5 recruits the Clr6-I histone deacetylase complex, which deacetylates histones at MBF-dependent gene promoters, thereby inducing transcriptional repression. Upon exit from quiescence (+Nitrogen), the levels of the MBF activator Rep2 increase, and Rep2 recruits the histone acetyltransferase SAGA complex,^18^ leading to activation MBF-dependent genes and promoting the G1/S transition.

To test if recruitment of HDACs to MBF promoters during quiescence results in decreased histone acetylation, we performed ChIP analysis of *whi5Δ*, *P41nmt1-whi5*^*+*^, and wild-type cells with an antibody that detects acetylation at lysines 9 and 14 of histone H3, which are targets of the Clr6-I complex.^73^^,^^74^ We found higher levels of acetylation at these residues in the *whi5Δ* mutant and reduced levels in the *whi5*^*+*^-overexpressing cells compared to the wild type at the promoters of *cnp1*^*+*^ and *hop1*^*+*^, whose expression is increased in the *whi5Δ* mutant, but also at the promoters of *cdc18*^*+*^ and *cdc22*^*+*^, whose expression is not significantly increased (Figures 6E; Table S1), suggesting that, besides Whi5/Clr6-dependent deacetylation, other mechanisms are contributing to their silencing. Taken together, our findings suggest that histone deacetylation contributes to silencing MBF-dependent early meiotic genes during quiescence, and that HDAC recruitment to these sites is mediated by the Whi5 repressor (Figure 7). However, mechanisms other than deacetylation ensure downregulation of critical targets whose untimely expression would pose a risk for cell viability.

## Discussion

In this study, we describe the role of the MBF transcriptional repressor Whi5 in fission yeast and demonstrate its contribution to the repression of MBF-dependent early meiotic genes during quiescence. In contrast to budding yeast, in *S. pombe*, the G1/S transition is regulated by a single transcription complex, MBF. This complex has been analyzed mainly under nutrient-rich conditions, where fission yeast cells spend most of their time in the G2 phase of the cell cycle and show a very short G1 phase and a cryptic G1/S cell size control. Under these conditions, MBF is active from mitosis onwards and adopts its repressor conformation in S-phase, associating with the co-repressors Nrm1 and Yox1, which are themselves targets of MBF.^22^^,^^23^^,^^24^^,^^25^^,^^27^ Under nitrogen starvation and in nitrogen-poor media (MMPhe and MMIle), when the G1 phase is extended, we detected the presence of the Whi5 inhibitor in the nucleus (Figures 3A-3D and 3G; S7), and both deletion and overexpression of *whi5*^*+*^ altered the dynamics of the G1/S transition. While overexpression of *whi5*^*+*^ showed a clear delay at the G1/S transition under nitrogen starvation, in nitrogen-poor medium and during exit from quiescence, we observed very subtle defects in the *whi5Δ* mutant when cells were shifted from nitrogen-rich to nitrogen-poor conditions, as well as a small but highly reproducible acceleration of the G1/S transition in *whi5Δ* cells during recovery from nitrogen starvation (Figures 1B–1D; S1). The subtle differences are probably due to the coexistence with other controls that ensure cell-cycle arrest in G1 during entry into quiescence, such as the inhibition of CDK1/Cyclin activity by the combined effects of the Rum1 CDK inhibitor and the Srw1/Ste9 APC/cyclosome activator.^75^^,^^76^^,^^77^^,^^78^ In addition, we have shown that the transcriptional repressor Nrm1 contributes to the repression of MBF-dependent expression during the cell divisions following nutritional shifts (Figure 4). Furthermore, according to its MBF-dependent expression, *nrm1*^*+*^ mRNA is upregulated in the *whi5Δ* mutant at 24 h in MM-N (Figures 2B; Table S1), suggesting that the higher levels of Nrm1 in the *whi5Δ* mutant could partially compensate for the absence of *whi5*^*+*^ under nitrogen starvation. Also, the levels of MBF components decrease during nitrogen starvation, which may contribute to MBF inactivation even in the absence of Whi5 (Figure 3F).

In previous work, we showed that in a nitrogen-poor medium (MMPhe), deletion of the MBF co-activator *rep2Δ* induces a marked increase in cell size, probably due to checkpoint activation triggered by replicative stress.^38^ This result demonstrated that proper activation of MBF is especially relevant for maintaining genome integrity in nitrogen-poor medium.^38^ In this study, we show that cells deleted for *res1*^*+*^ (*res1Δ*) also exhibit this phenotype in nitrogen-poor medium (Figure S2B). In addition, *res1Δ* cells display slow growth in nitrogen-rich medium, as this gene is quasi-essential (Figure S2A). We observed that the increased cell size of *res1Δ*, *rep2Δ,* and *cdc10-129* mutants in nitrogen-poor medium and the slow growth of *res1Δ* in nitrogen-rich medium are significantly suppressed by *whi5*^*+*^ deletion^47^^,^^48^ (Figures S2A-S2D). Furthermore, under nitrogen-poor conditions, overexpression of *whi5*^*+*^ also induces a G1 delay, increased DNA damage, and increased cell size, mimicking the phenotype of MBF mutants (Figures 1D and S1B and S1C). Interestingly, very subtle defects occur when *whi5*^*+*^ is overexpressed in nitrogen-rich medium (MM; Figures 1B–1D). Only cells with a prolonged G1 phase, such as those growing in nitrogen-poor conditions or under nitrogen starvation, are sensitive to *whi5*^*+*^ overexpression, perhaps because Whi5 is a specific inhibitor of MBF complexes composed of Cdc10 and Res2, which are the main components of MBF under these conditions. Supporting this hypothesis, neither deletion nor overexpression of *whi5*^*+*^ had any effect on the *res2Δ* mutant (Figure S2E; S3C–S3E). Furthermore, the slow-growth phenotype of the *res1Δ* mutant in nitrogen-rich medium, where START is entirely dependent on the Res2-Cdc10 complexes, is strongly suppressed by the deletion of *whi5*^*+*^, and overexpression of *whi5*^*+*^ in *res1Δ* induces cell size elongation even in nitrogen-rich medium (Figures S2A and S3A).

The extension of G1 in *S. pombe* is physiologically associated with worsening nutritional conditions, a situation that is in fact most common for cells in nature, where nutrient availability is an ever-changing factor and determines cell size at the time of division. Under these conditions, the presence or absence of a mating partner determines whether cells with an extended G1 phase proceed to undergo mating, meiosis, and sporulation or enter a quiescent state. During meiosis, the MBF complex has been shown to be essential for the expression of premeiotic S-phase and recombination genes.^15^^,^^51^^,^^52^ Interestingly, this complex is composed of Cdc10 and Res2, with Rep1 replacing Rep2 as a coactivator during meiosis.^79^^,^^80^ In this study, we observed that the wave of premeiotic S-phase and recombination genes is upregulated in quiescent *whi5Δ* cells, suggesting that the Whi5 inhibitor targets this subset of genes, contributing to their repression under nitrogen starvation conditions until pheromone signals induce meiosis (Figure 2; Tables S1 and S2). Regulation in fission yeast thus contrasts with that in budding yeast, where the wave of early meiotic genes is induced by the Ime1 transcription factor, and where proper activation of Ime1 requires inhibition of the SBF complex, partly mediated by Whi5.^81^^,^^82^^,^^83^ These differences can be explained by differences in SBF and MBF in budding yeast. While SBF is required for budding and morphogenesis only during mitotic growth, MBF is required for the activation of DNA replication and repair genes, functions necessary not only during mitotic growth but also in early meiosis.^83^^,^^84^^,^^85^

Our study suggests that histone deacetylases (HDACs) may play a crucial role in the entry and maintenance of quiescence in fission yeast. In *S. pombe*, the histone deacetylase Clr6 (orthologue of budding yeast Rpd3 and human HDAC1/2) is present in two complexes, Clr6-I and Clr6-II. The essential Clr6-I complex, containing the Pst1 and Pst3 subunits, preferentially deacetylates gene promoters and regulates transcription of protein-coding genes. In contrast, the non-essential Clr6-II complex, which contains the specific Pst2 subunit, targets coding regions and prevents cryptic transcription.^66^^,^^67^^,^^68^ We observed that Whi5 interacts with the Clr6-I complex during quiescence, suggesting a potential role in the downregulation of specific gene subsets, including early meiotic genes, which may be regulated by the MBF complex in quiescence (Figure 6; Table S3). Supporting our findings, a proteomic analysis of Clr6 complexes in *S. pombe* by Wang et al. (2023) identified components of the MBF complex among the interactors of the Pst3 subunit of the Clr6-I complex, but not among the interactors of the Pst2 subunit of the Clr6-II complex, in rich medium.^67^ Consistent with the role of acetylation in MBF-dependent expression in fission yeast, the SAGA complex has been shown to be necessary for the proper induction of G1/S transcripts in mitotically dividing cells in rich medium.^18^ Furthermore, a genetic screen in *S. pombe* aimed at identifying genes required for maintaining viability during cell quiescence identified the Clr6 complex, the MBF repressor Yox1, and the co-repressors Nrm1 and Whi5, suggesting that the repression of MBF target genes is essential in G0 to preserve viability.^12^

In budding yeast, the HDAC Rpd3, a homologue of fission yeast Clr6, induces a global shutdown of gene expression during quiescence.^3^^,^^4^^,^^13^^,^^86^ However, in fission yeast, there are no reports suggesting a role for Clr6 in establishing heterochromatin during quiescence, as this function depends on histone methylation and RNA interference.^2^^,^^5^^,^^6^ In line with the role of budding yeast Rpd3 in the transition from proliferation to quiescence, a recent study suggests that, although Rpd3 shows a limited role in regulating G1/S transcript levels in proliferating cells, it is required for complete repression in G1 cells.^87^ Furthermore, Rpd3 is recruited by the Ume6 repressor to silence early meiotic genes in mitotically dividing cells.^88^^,^^89^^,^^90^^,^^91^ Similarly, in *S. pombe*, the HDAC Clr6 plays an important role in repressing meiotic and environmental stress genes.^74^^,^^92^ In line with our observations in fission yeast, both budding yeast Whi5 and human RB have been shown to recruit HDACs to the promoters of SBF- and E2F-regulated genes, respectively, with deacetylation contributing to gene repression.^69^^,^^70^^,^^71^^,^^93^^,^^94^^,^^95^^,^^96^

Finally, an intriguing observation regarding Whi5 localization is that, although Whi5 is mainly a nuclear protein during quiescence, a fraction remains in the cytoplasm and, according to our mass spectrometry analysis, may interact with mitochondria or the endoplasmic reticulum (Figure S9; Table S3). Interestingly, in *S. cerevisiae*, Whi5 has a paralog, Whi7, which is located not only in the nucleus but also in the endoplasmic reticulum, where it can control the G1/S transition by retaining G1/S cyclins, independently of transcription.^32^ In *S. pombe*, the function of this cytoplasmic fraction of Whi5 remains to be elucidated. Moreover, in budding yeast, Whi7 exhibits a specific function not shared with ScWhi5 in the cell wall stress response,^30^^,^^31^ and, along with ScWhi5, it also regulates the entry into quiescence.^33^^,^^34^^,^^35^ Our findings in fission yeast support the notion of a conserved role of these repressors in quiescence and highlight the utility of *S. pombe* as a model organism for investigating novel aspects of G1/S transcriptional regulators, such as the tumor suppressor RB, in the cell cycle and in the transition to quiescence.

### Limitations of the study

Our study demonstrates that Whi5 is essential for repressing a subset of MBF-dependent genes during quiescence by recruiting the Clr6-I complex to their promoters. Although our findings strongly support a functional connection between MBF and Clr6-I complexes mediated by Whi5, further investigation is needed to establish a direct link and fully elucidate the underlying mechanism. Transcriptome analysis of conditional mutants of the Clr6-I complex, compared with the *whi5Δ* mutant during quiescence entry, could provide additional support for our model. Moreover, complementary approaches would help to further characterize this mechanism. For instance, performing ChIP-seq for Pst3 in MM-N conditions would allow us to confirm the presence of the Clr6-I complex at the promoters of genes deregulated in the *whi5Δ* mutant.

## Resource availability

### Lead contact

Further information and requests for resources and reagents should be directed to and will be fulfilled by the lead contact, Sergio Moreno (smo@usal.es).

### Materials availability

Strains generated in this study are available from the lead contact upon request.

### Data and code availability

• RNA-Seq data have been deposited at GEO (https://www.ncbi.nlm.nih.gov/geo/) with accession numbers GSE285081 and GSE285763 and are publicly available as of the date of publication.
• Proteomics data have been deposited at PRIDE (https://www.ebi.ac.uk/pride/) with identifiers PXD057838 and PXD057886 and are publicly available as of the date of publication.
• All data reported in this article will be shared by the lead contact upon request.
• This article does not report any original code.
• Any additional information required to reanalyse the data reported in this article is available from the lead contact upon request.

## Acknowledgments

The authors thank Victor A. Tallada for plasmids and advice on the use of the BiFC system, and Patricia García and Alicia Vázquez-Bolado for their help with the ChIP experiments. Carmen Castro and Rebeca Martín for microscopy assistance (IBFG Microscopy Service), Mar Sánchez for technical assistance in RNA preparation for sequencing (IBFG Genomic Service), Jesús Pinto for image analysis assistance (IBFG Bioinformatics Service), and Rosa Degano and Nieves Ibarrola for their technical assistance in mass spectrometry analyses (Salamanca Cancer Research Institute Proteomic Facility). Ana Elisa Rozalén for her technical support, Javier Encinar for his helpful suggestions, and all members of the Moreno lab for their valuable discussions and comments on the manuscript. This work was funded by the Spanish Ministry of Science and Innovation-MCIN (grants BFU2017-88335-R, PID2020-115929RB-I00 and PID2023-146972NB-I00) and from the Castile and Leon government (grants CSI259P20, CSI010P23 and IBFG Unit of Excellence programs CLU-2017-03 and CL-EI-2021-08 co-funded by the P.O. Feder of Castile and Leon 14–20 and European Union ERDF “Europe drives our growth”). C.G.-M. is a recipient of a predoctoral FPU contract from the MUNI (FPU19/04115). R.L.-S.S. is funded by a predoctoral contract from the Castile and Leon government. D.G.-A. was funded by a predoctoral FPI contract from the MICIU (PRE2018-084956). L.P.-H. is a recipient of a postdoctoral contract from the Castile and Leon government.

## Author contributions

Conceptualisation: C.G.-M., J.A., L.P.-H., and S.M.; formal analysis, investigation, and methodology: C.G.-M., R.L.-S.S., M.B.S., D.G.-A., and L.P.-H.; visualization: C.G.-M., R.L.-S.S., M.B.S., and L.P.-H.; writing – review and editing: C.G.-M., R.L.-S.S., M.B.S., D.G.-A., J.A., L.P.-H., and S.M.; writing – original draft: L.P.-H.; supervision, funding acquisition, and project administration: S.M.

## Declaration of interests

The authors declare no competing interests.

## STAR★Methods

### Key resources table

**Table:** 

REAGENT or RESOURCE	SOURCE	IDENTIFIER
**Antibodies**		

Mouse monoclonal anti-myc	Sigma	Cat# M5546; RRID:AB_260581
Mouse monoclonal anti-mini-AID-tag	MBL	Cat# M214-3; RRID:AB_2890014
Mouse monoclonal anti-HA	Roche	Cat# 11666606001; RRID:AB_514506

**Chemicals,peptides,andrecombinantproteins**		

TB Green Premix Ex Taq (Tli RNAse H Plus)	Takara	RR420A
SuperScript First-Strand Synthesis System for RT-PCR	Invitrogen	11904018
DNAse I	Invitrogen	18068015
Expand High Fidelity PCR System	Roche	11732650001
5-adamantyl-IAA	Tokyo Chemical Industry	A3390
Complete Protease Inhibitor Cocktail	Merk	11697498001
GFP-Trap Magnetic Agarose	ChromoTek	gtma-100
Trichloroacetic Acid (TCA)	Merk	100807
Formaldehyde	Merk	104003
L-Phenylalanine	Merk	1072560100
L-Isoleucine	BioChemica	A3677
Propidium iodide	Merk	P4170

**Criticalcommercialassays**		

Clarity Western ECL Substrate	Bio-Rad	170–5061

**Depositeddata**		

RNAseq data (GEO repository, NCBI; https://www.ncbi.nlm.nih.gov/geo/)	This study	GEO: GSE285081
RNAseq data (GEO repository, NCBI; https://www.ncbi.nlm.nih.gov/geo/)	This study	GEO: GSE285763
Proteomics data (PRIDE repository, EMBL-EBI; https://www.ebi.ac.uk/pride/)	This study	PRIDE: PXD057838
Proteomics data (PRIDE repository, EMBL-EBI; https://www.ebi.ac.uk/pride/)	This study	PRIDE: PXD057886

**Experimentalmodels:Organisms/strains**		

*S. pombe* strains are listed in Table S5	N/A	N/A

**Oligonucleotides**		

See Table S6	N/A	N/A

**Software andalgorithms**		

R software	N/A	https://www.r-project.org/
DESeq2 v1.22.2	Love et al.^97^	https://bioconductor.org/packages/release/bioc/html/DESeq2.html
FastQC v0.11.8	Babraham Bioinformatics	https://www.bioinformatics.babraham.ac.uk/projects/fastqc/
Trimmomatic v0.38	Bolger et al.^98^	http://www.usadellab.org/cms/index.php?page=trimmomatic
HISAT2 V2.2.0	Kim et al.^99^	https://www.ccb.jhu.edu/software/hisat/index.shtml
Samtools V1.9	Li et al.^100^	https://www.htslib.org/
featureCounts (subread package v1.6.3)	Liao et al.^101^	https://subread.sourceforge.net/
String v12.0	Szklarczyk et al.^102^	https://string-db.org/
ImageJ Fiji	Schindelin et al.^103^	https://fiji.sc/
CellQuest Pro 6.0.3	Becton Dickinson Biosciences	https://www.bdbiosciences.com/
Fusion 2.2	Oxford Instruments Andor	https://andor.oxinst.com/
SoftWoRx 5.5.0	Cytiva	https://www.cytivalifesciences.com/
MetaMorph 7.7.8	Molecular Devices	https://www.moleculardevices.com/
Excel 16.66.1	Microsoft	https://www.microsoft.com/
GraphPad Prism 10	GraphPad Software Inc.	https://www.graphpad.com/

### Experimental model and study participant details

#### Fission yeast strains and growth conditions

The strains and oligonucleotides used in this study are listed in Tables S5 and S6, respectively. Standard protocols were followed for the growth and manipulation of fission yeast cells.^104^ Cells were cultured in Edinburgh minimal medium containing 93.5 mM ammonium chloride (EMM2, referred to as MM in the text and figures) at the indicated temperatures.

Exponentially growing cells were cultured in MM and then transferred to minimal medium without NH_4_Cl (MM-N) at 25°C for nitrogen starvation experiments or to MM-N supplemented with 20 mM phenylalanine (MMPhe) or 20 mM isoleucine (MMIle) at the indicated temperature for nutritional shift-down experiments. To release cells from the nitrogen starvation block, NH_4_Cl was added from a 2 M stock solution to a final concentration of 93.5 mM, and the cultures were shifted to 32°C.

For expression from the *nmt1* promoter, cells were grown to logarithmic phase in minimal medium containing 5 μg/mL thiamine (promoter OFF), washed three times with minimal medium without thiamine, and then inoculated into the same medium at the indicated temperature and for the specified time (promoter ON).^105^

For conditional depletion of Nrm1, the degron system described by Zhang et al. (2022) was used.^62^ To induce Nrm1 degradation, 100 nM 5-adamantyl-IAA was added to minimal medium from a 1 mM stock solution (Tokyo Chemical Industry, A3390).

### Method details

#### Yeast strain generation

The methods described in Bähler et al. (1998) were used to tag genes at the C-terminus with GFP, YFP, 13myc, nYFP, cYFP, or 3xsAID and to introduce the *nmt1*^*+*^ promoter at the *whi5*^*+*^ locus.^106^ A strain containing the Padh1-OsTIR1-F74A cassette at the *ura4*^*+*^ locus was transformed to tag the *nrm1*^*+*^ gene with the 3xsAID epitope at the C-terminus.

#### Flow cytometry analysis

Cells were fixed in 70% (v/v) ethanol, washed with 50 mM sodium citrate, and resuspended in the same solution containing 0.1 mg/mL RNAse A. After overnight incubation at 37 °C, the cell suspensions were mixed with a solution containing 4 μg/mL propidium iodide and 50 mM sodium citrate, and sonicated.^107^ Samples were analyzed using a Becton-Dickinson FACScan flow cytometer equipped with CellQuest software.

#### Microscopy

BiFC experiments and analysis of Whi5 and Nrm1 localization were performed using an Andor Dragonfly 200 spinning disk confocal microscope equipped with a 60x/NA 1.40 Oil objective and an Andor Sona sCMOS 4.2B-11 camera, controlled by Fusion software. A stack of 8 confocal images with a Z-step of 0.2 μm was taken. Images were deconvolved (except in Figures 3G and 6C) and projected into a 2D image with the maximum intensity projection setting. To visualise Rad52-YFP foci, a DeltaVision microscope system equipped with an Olympus IX71 inverted microscope, a 60x Plan Apo 1.42 objective, a Photometrics CoolSNAP HQ2 camera, and SoftWoRx software (Cytiva) was used. Stacks of 12 images with a spacing of 0.3 μm were captured, and maximum projections were generated. The percentage of cells with one or more foci was analyzed for each strain and condition in two independent experiments, with the means and standard deviations shown. For cell size measurements, live cells were stained with 50 μM calcofluor in PBS and examined under a Nikon Eclipse 90i fluorescence microscope equipped with a 60x/1.40 Oil Plan Apo objective and a Hamamatsu ORCA-ER camera. Live images were acquired using MetaMorph software (Molecular Devices). At least 300 cells were measured for each strain and timepoint. To analyze Whi5-GFP localization, the coefficient of variation across the cell area was used as a readout for nuclear localization. After generating a sum projection of Z stacks (8 sections, 0.2 step size) and applying a mean filter (radius 1), a cell mask was generated using a grayscale Otsu threshold. The resultant mask was then applied to the original projection to measure the mean gray values and standard deviations. To confirm that the cytoplasmic signal of Whi5-GFP is above the background signal of an untagged strain, a quantification was performed by drawing areas in the cytoplasm and nuclear regions in *whi5-GFP* and untagged cells using the sum projections. The mean gray values of each area were measured, and the intensity values from areas outside the cells were subtracted from all images. The resulting signal intensities were then used to measure the ratio between the signals in the *whi5-GFP* and the untagged strain. All images were processed and assembled using Fiji software.^103^

#### Protein extractions and western blots

Protein extractions were performed using the TCA extraction protocol described by Sansó et al. (2008).^108^ Briefly, 10 mL of culture with an O.D._595_ of 0.5 was treated with TCA to a final concentration of 10%. Cells were washed with 20% TCA, and the resulting pellets were resuspended in 0.1 mL of 12.5% TCA. Cell lysis was carried out in the presence of glass beads using a Fast-Prep FP120 cell disruptor (BIO101) with 3 pulses of 20 s at power setting 5.5. Lysates were collected in new 1.5 mL tubes and centrifuged at 13200 rpm for 20 min at 4 °C. The supernatants were discarded, and the pellets were washed with 1 mL acetone and dried in a thermoblock at 55 °C for 10 min. Protein extracts were then resuspended in a solution containing 1% SDS, 100 mM Tris-HCl pH 8 and 1 mM EDTA. The samples were loaded on 8% SDS-PAGE gels, transferred to PVDF Immobilon P membranes (Millipore), and probed with the following primary antibodies: anti-Myc (1:3000 dilution; Sigma), anti-HA (1:3000 dilution; Roche), anti-AID (1:2000 dilution; MBL) and anti-actin (1:1000 dilution; Santa Cruz). Secondary antibodies used were goat anti-mouse (1:2500 dilution; NA 931, Amersham) and goat anti-rabbit (1:3000 dilution; NA 934, Amersham), both conjugated to horseradish peroxidase. Membranes were developed with ECL western blot reagents (Clarity, BioRad) and blots were quantified with Fiji software.

#### Immunoprecipitations and mass spectrometry analysis

Immunoprecipitations were performed following the protocol described by García et al. (2021), with some modifications.^109^ Purifications were carried out with 1 L of cells for Cdc10-L-YFP and 1.6 L for Whi5-GFP. Cells were crosslinked with 1% formaldehyde for 10 min at 25 °C. After quenching with 250 mM glycine and incubating on ice for 5 min, cells were collected, washed twice with cold PBS buffer, and lysed using a freezer mill (Cole-Parmer) in lysis buffer containing 25 mM Tris HCl pH 7.5, 150 mM NaCl, 0.5% SDS, 1% Igepal, 10 mM PMSF, 1 μg/mL aprotinin, 1 μg/mL leupeptin, 20 μM bortezomib, and Complete protease inhibitor cocktail (Sigma). Before clarifying the protein extracts, the SDS concentration was reduced to 0.1% by diluting with lysis buffer lacking SDS.

GFP- or YFP-tagged proteins were immunoprecipitated using 30 μL of GFP-trap magnetic beads (Chromotek) for 1 h at 4 °C. The beads were washed six times with lysis buffer containing 500 mM NaCl and twice with 100 mM Tris HCl pH 8.5 before mass spectrometry analysis. Mass spectrometry was performed at the Proteomics Facility of the Salamanca Cancer Research Center, and data analysis was carried out using the String and Shinygo webtools.^102^^,^^110^

#### RNA methods

For RNAseq and qPCR analyses, samples of 2x10^8^ cells cultured in MM or MM-N were collected at the indicated time points, washed with 1 mL of cold water, and frozen in dry ice. Total RNA was extracted using TRIzol Reagent (Invitrogen) following the manufacturer’s instructions. RNA integrity was checked by electrophoresis, and RNA concentration was measured with a Nanodrop microspectrophotometer.

For qPCR analysis, RNA samples were treated with RNAse-free DNAse I (Invitrogen) for 30 min at 25 °C. Then, 1–2 μg of RNA was reverse transcribed using the SuperScript First-Strand Synthesis System (Invitrogen) with oligo(dT) at 50 °C for 30 min. cDNA (1 μL) was used for qPCR amplification in an Applied Biosystems 7300 Real-Time PCR System with the following cycling conditions: 95 °C for 45 s, followed by 40 cycles of 95 °C for 5 s and 60 °C for 31 s. The amplification was completed with a dissociation step consisting of 95 °C for 15 s, 60 °C for 1 min and 95 °C for 15 s. Reactions were performed in a final volume of 20 μL using TB Green Premix Ex Taq (TaKaRa) and oligonucleotides at a concentration of 0.2 μM (Table S6). Reactions were carried out in duplicate or triplicate, and negative controls without cDNA, reverse transcriptase or oligonucleotides were included in each run to control DNA contaminations. Actin was used for initial normalisation, and fold changes relative to the wild type grown in MM were calculated using the method described by Pfaffl et al. (2001).^111^ At least two biological replicates were performed for each experiment.

For RNAseq analysis, two biological replicates were sequenced for each timepoint. Library preparation and subsequent NGS analysis were performed by Novogene. Libraries were prepared from total RNA using polyA enrichment of the mRNA. Sequencing was performed in an Illumina NovaSeq X plus platform using a paired-end protocol. Sequence quality was assessed with FastQC (v 0.11.8, Babraham Bioinformatics). Adapters were removed from sequencing reads using Trimmomatic (v 0.38) if necessary.^98^ Reads were aligned to the reference genome using HISAT2 (v 2.1.0, CCB, John Hopkins University).^99^ The reference genome was downloaded from Pombase.org^112^(downloaded on 31-10-2018). Samtools (v 1.9) was used to manipulate and process DNA sequence read alignments. Alignment files were converted to BigWig format with bamCoverage (deepTools v 3.3.0). BigWig files were used to visualise alignments in IGV (v 2.4.16) and JBrowse (v 1.15.4). The number of reads associated with genomic features was quantified using featureCounts (Subread package version 1.6.3, Walter+Eliza Hall Bioinformatics).^101^ Statistical analysis of read counts was performed with R software. Bioconductor DESeq2 package (v 1.22.2) was used for differential expression analysis.^97^ Heatmaps were generated with Pheatmap (v 1.0.12). Differentially expressed transcripts were defined as transcripts that increased >2^0.^^585^ (approximately equal to 1.5-fold) or decreased <2^−0.585^ (approximately equal to 0.66-fold), with adjusted *p* - value<0.001. The Angeli and the Shinygo web tools were used for enrichment analyses.^110^^,^^113^

#### Chromatin immunoprecipitation

Chromatin immunoprecipitation was performed with a 200 mL culture of quiescent cells (0.5–0.6 O.D._595_). Wild type, *whi5Δ* and *P41nmt1-whi5*^*+*^ cells were cultured in YES medium to mid-exponential phase and then transferred to MM with thiamine (MMT, *nmt1* promoter OFF). After 24 h of growth in MMT, cells were washed and shifted to MM without thiamine (*nmt1* promoter ON). The *whi5*^*+*^ gene was induced from the *nmt1* promoter for 24 h in MM-T before shifting cells to MM-N to induce entry into quiescence. After 16 h in MM-N, cells were fixed with 1% of formaldehyde (Sigma) for 10 min at 25 °C with agitation. The fixation reaction was quenched by adding glycine to a final concentration of 125 mM, and cultures were then incubated on ice for 5 min. Cells were centrifuged at 4000 rpm for 5 min at 4 °C, washed twice with 10 mL of cold PBS, frozen in dry ice, and stored at −80 °C.

Pellets from 200 mL of culture were resuspended in 0.5 mL of lysis buffer (0.1 M Tris-HCl pH 8, 20% glycerol, supplemented with 10 mM PMSF -Sigma-), and lysed in the presence of glass beads (50 μm; Sigma) using a Fast-Prep FP120 cell disruptor (BIO101). Lysis was carried out with 2 cycles of 45 s at power setting 4.5, with a 2-min incubation on ice between cycles. The tubes were then punctured, and the extracts were recovered in new 1.5 mL tubes by centrifugation at 7000 rpm for 10 s. The extracts were centrifuged at 13200 rpm for 1 min at 4 °C, and the precipitates were washed with 1 mL of lysis buffer (50 mM HEPES pH 7.6 -Sigma-, 140 mM NaCl, 1 mM EDTA, 1% Triton X-100 -Sigma-, 0.1% sodium deoxycholate -Sigma-, 0.1% SDS, supplemented with 10 mM PMSF). Pellets were resuspended in 0.5 mL of lysis buffer and lysates were divided into two 0.25 mL aliquots and sonicated with a Bioruptor PIco (Diagenode) using 6 cycles of 30 s ON and 30 s OFF to generate DNA fragments approximately 500 bp in size. After sonication, 750 μL of lysis buffer were added, and extracts were centrifuged at 13200 for 30 min at 4 °C. The supernatants were collected, and 50 μL of each strain sample was reserved and stored at −20 °C as ‘input’. The remaining samples were divided into 2 tubes, and 1 μL of either anti-H3 (ref. ab1791 Abcam), or anti-H3K9K14 (ref. 06–599; Sigma) antibodies were added to each tube. Protein G-coupled magnetic beads (Dynabeads Protein G, Thermo Fisher), previously equilibrated in lysis buffer, were then added. The samples were incubated overnight at 4 °C with rotation. The following day, beads were washed as follows: once with 1 mL of lysis buffer, twice with 1 mL of lysis buffer containing 500 mM NaCl, twice with 1 mL of wash buffer (10 mM Tris-HCl pH 8, 1 mM EDTA, 250 mM LiCl, 0.5% sodium deoxycholate, 0.5% Igepal -Fluka-, 1 mM PMSF), and once with 1 mL of TE buffer. For each wash, samples were incubated at room temperature for 5 min with rotation. Immunoprecipitations were then eluted with 100 μL of elution buffer (50 mM Tris-HCl pH 8, 10 mM EDTA, 1% SDS) for 20 min at 65 °C, and the eluates were transferred to a new tube. Beads were washed again with 150 μL of TE buffer with 0.67% SDS, and both eluates were pooled. The previously separated inputs were thawed, and 150 μL of TE buffer with 0.67% SDS was added. Both immunoprecipitations and inputs were incubated at 65 °C overnight to reverse crosslinking. The next day, 250 μL of TE buffer containing 1 μL of 20 mg/mL glycogen and 7.5 μL of 20 mg/mL proteinase K were added, and samples were incubated at 37 °C for 2 h. DNA was extracted with phenol-chloroform and precipitated with 100% ethanol for at least 4 h at −20 °C. Samples were centrifuged at 13200 rpm for 30 min at 4 °C. The supernatants were carefully removed, and the pellets were washed with 1 mL of 70% ethanol at −20 °C. The pellets were air-dried for 30 min, resuspended in 100 μL of TE buffer, and stored at −20 °C.

Purified DNA was used as template for qPCR amplification with oligonucleotides targeting the promoter of the gene analyzed (Table S6). PCR reactions were performed in a final volume of 10 μL with 1 μL of DNA, 5 μL of TB Green Premix Taq (Tli RNase H Plus, TaKaRa), and 0.2 μM oligonucleotides. Amplification was carried out using a Bio-Rad CFX96 thermal cycler with the following protocol: 95 °C for 30 s, followed by 40 cycles of 95 °C for 5 s and 60 °C for 30 s. This was followed by a dissociation step with 95 °C for 30 s, 65 °C for 35 s, and 95 °C for 50 s. Reactions were performed in duplicate, with negative controls lacking DNA included in each run. Data were normalised to actin and input DNA, and the H3K9K14ac/H3 ratio was calculated. The average ratio and standard deviation of two biological replicates of the experiment was reported.

### Quantification and statistical analysis

Graphs and statistical analysis were performed using Excel (Microsoft 365) or Prism GraphPad (version 10) software. A two-tailed Student’s *t* test was used for comparisons between two groups in Figure 2E. Cell size datasets were assessed for normality using the Shapiro-Wilk test. In these cases, datasets were not normally distributed, and comparisons were performed with a non-parametric Kruskal-Wallis test with Dunn’s correction. To calculate the statistical significance of the overlap of two sets of genes (Figure 2B) a hypergeometric test was performed with the online tool http://nemates.org/MA/progs/overlap_stats.html, considering a total of 5134 codifying genes. Tests used are indicated in the figure legends.

## Supplemental information

Document S1. Figures S1–S10 and Tables S5 and S6

Table S1. RNAseq analysis of whi5Δ vs. wild type, related to Figure 2 Differentially expressed genes in *whi5Δ* versus the wild type in MM, 10 h MM-N and 24 h MM-N. Upregulated genes: adjusted p-value<0.001, fold change>2^0.^^585^; Downregulated genes: adjusted p-value<0.001, fold change<2^−0.585^.

Table S2. Enrichment analysis of genes upregulated in whi5Δ at 24 h in MM-N performed with the AnGeLi web tool, related to Figure 2

Table S3. Mass spectrometry results of Whi5-GFP, related to Figures 5 and 6 Proteins interacting with Whi5-GFP in MM and MM-N (16 h).

Table S4. Mass spectrometry results of Cdc10-L-YFP, related to Table 1 Proteins interacting with Cdc10-L-YFP in MM-N (16 h) in *whi5*^*+*^ and *whi5Δ* cells.
