# Supplementary material for: Fission yeast Whi5 represses MBF-dependent transcription in quiescent cells
Source: iScience. 2025 Dec 30;29(2):114576. doi: 10.1016/j.isci.2025.114576 (PMC12828573; doi:10.1016/j.isci.2025.114576)
Supplement: Document S1. Figures S1–S10 and Tables S5 and S6 [file mmc1.pdf]

iScience, Volume *29*

Supplemental information

Fission yeast Whi5 represses MBF-dependent transcription in quiescent cells

Celia Gálvez-Merchán, Rafael López-San Segundo, M. Belén Suárez, Daniel González-Álvarez, José Ayté, Livia Pérez-Hidalgo, and Sergio Moreno

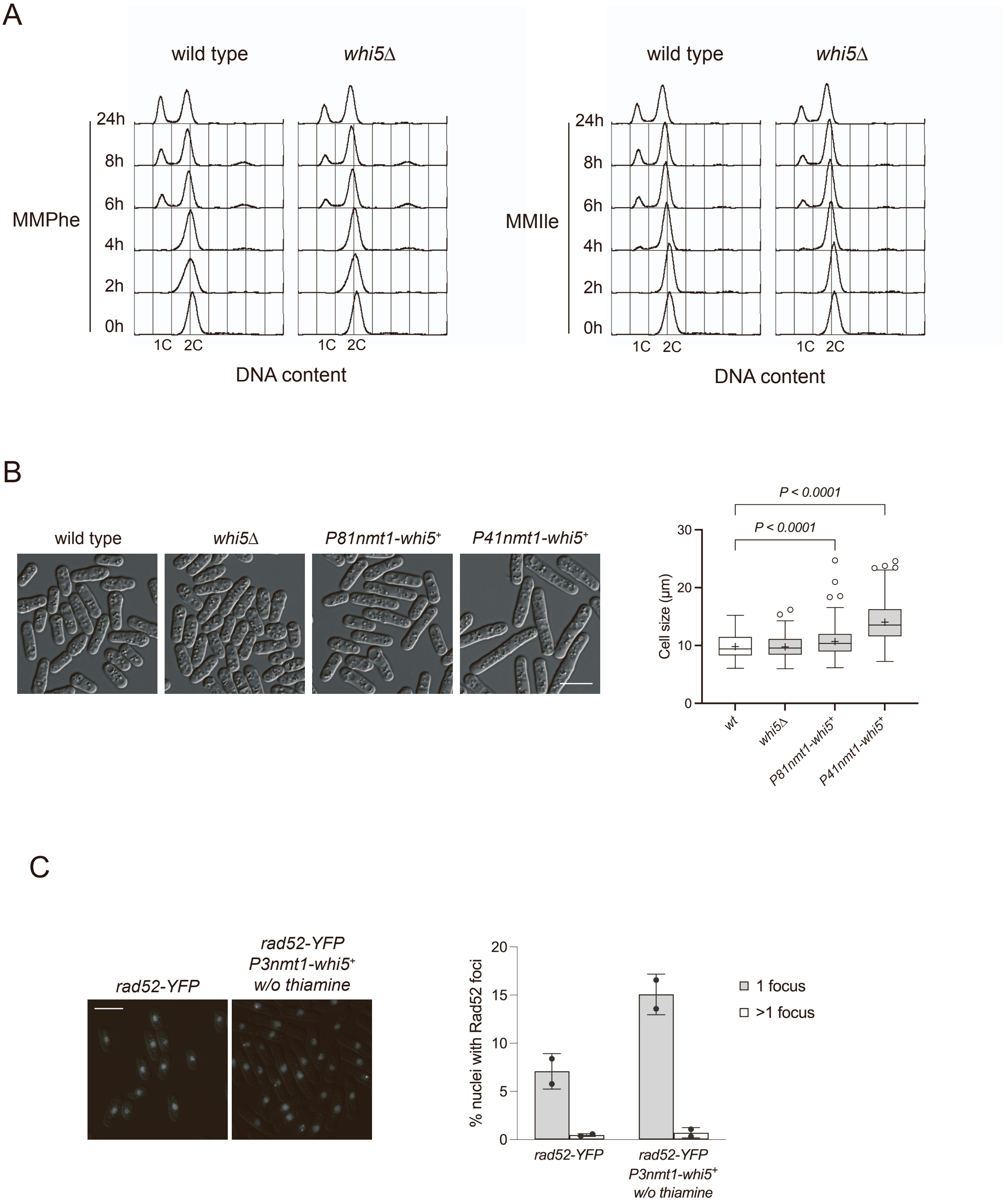
A
wild type
whi5Δ
24h
8h
6h
4h
2h
0h
MMPhe
MMIle
1C 2C
DNA content
B
wild type
whi5Δ
P81nmt1-whi5+
P41nmt1-whi5+
P < 0.0001
P < 0.0001
Cell size (µm)
30
20
10
0
wt
whi5Δ
P81nmt1-whi5+
P41nmt1-whi5+
C
rad52-YFP
rad52-YFP
P3nmt1-whi5+
w/o thiamine
% nuclei with Rad52 foci
20
15
10
5
0
1 focus
>1 focus

Figure S1. Whi5 levels regulate the G1/S transition in nitrogen-poor media (MMPhe and MMIle). Related to Figure 1.

(A) Flow cytometry analysis showing the DNA content of wild type and *whi5Δ* cells grown in minimal medium with phenylalanine (left panels) or isoleucine (right panels) at 25ºC. Cells were grown in MM, washed twice with MMPhe or MMIle, and incubated in the same media for 24 hours. Samples were collected at the indicated time points and analysed by flow cytometry. **(B)** Left panels: DIC images of wild-type, *whi5Δ*, *P81nmt1-whi5*$^{+}$ and *P41nmt1-whi5*$^{+}$ cells from Figure 1D after 24 hours of growth in MMPhe at 32 ºC. Right panel: cell size quantification. At least 300 calcofluor-stained cells from one representative experiment were measured. The data are represented with Tukey box-and-whisker plots, where the bars within the boxes represent the median, the crosses within the boxes represent the mean, and the upper and lower bounds of the boxes represent the 75th and the 25th percentiles, respectively. The whisker boundaries represent the upper value below the 75th percentile + 1.5 times IQR (interquartile range) and the lower value above the 25th percentile - 1.5 IQR, respectively. Each point above or below these limits are outliers, represented by symbols. Comparisons with the wild type were performed using the Kruskal-Wallis test (one-way, non-parametric ANOVA) with Dunn's correction for multiple comparisons. **(C)** Analysis of Rad52-YFP foci in wild-type and *P3nmt1-whi5*$^{+}$ strains grown in MMPhe for 20 hours at 32 ºC. Left panel: Fluorescence images of Rad52-YFP foci. Overlays with DIC images are shown. Right panel: Percentage of cells with one (light grey) or more than one nuclear focus (white) of Rad52-YFP. Data represent the means and standard deviations from two experimental replicas, with at least 500 cells analysed per strain in each experiment. Scale bars: 10 µm.

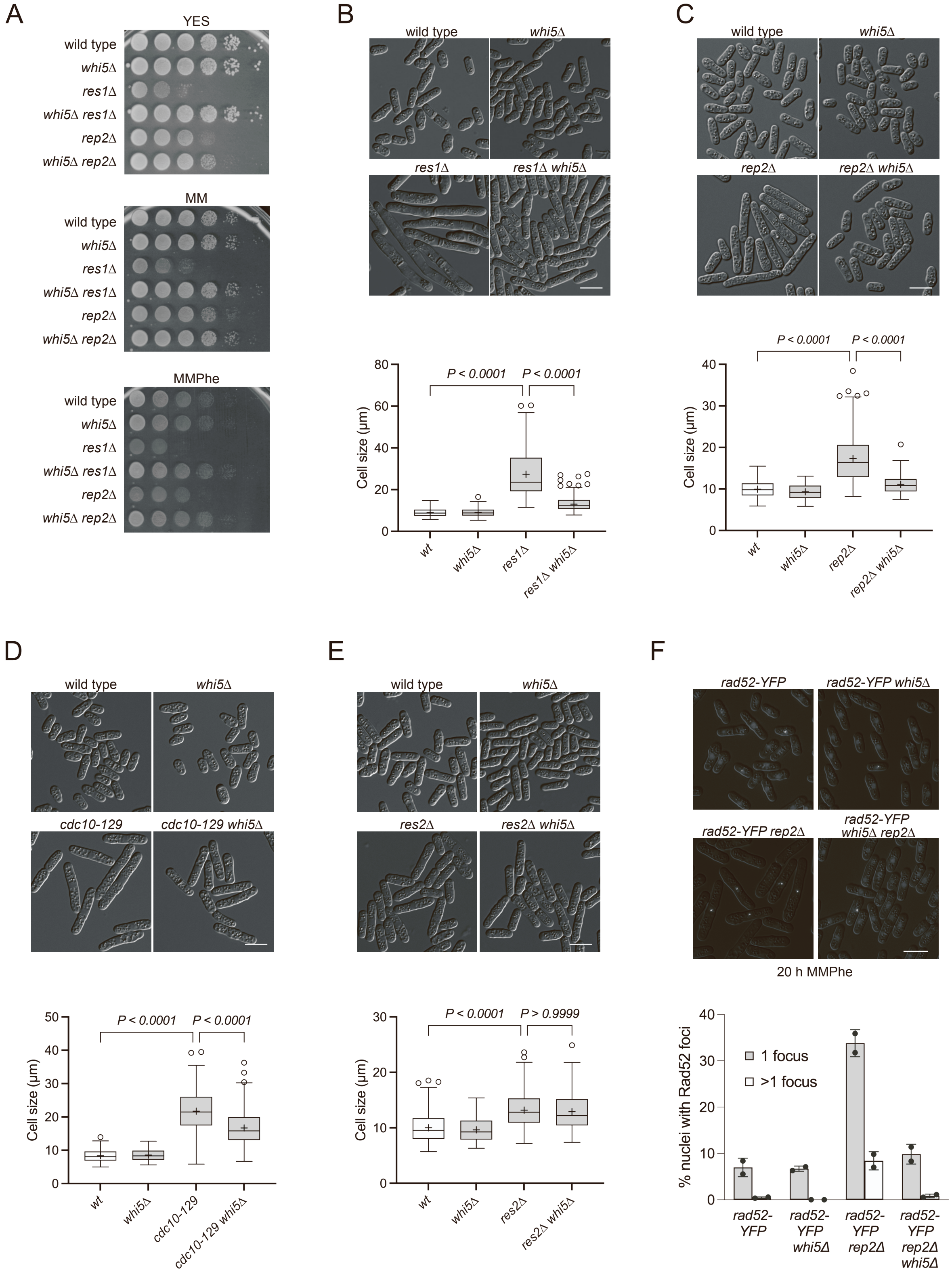
A
YES
wild type
whi5Δ
res1Δ
whi5Δ res1Δ
rep2Δ
whi5Δ rep2Δ
MM
MMPhe
B
wild type
whi5Δ
res1Δ
res1Δ whi5Δ
P < 0.0001
P < 0.0001
Cell size (μm)
wt
res1Δ whi5Δ
C
rep2Δ
rep2Δ whi5Δ
D
cdc10-129
cdc10-129 whi5Δ
E
res2Δ
res2Δ whi5Δ
P > 0.9999
F
rad52-YFP
rad52-YFP whi5Δ
rad52-YFP rep2Δ
rad52-YFP whi5Δ rep2Δ
20 h MMPhe
% nuclei with Rad52 foci
1 focus
>1 focus

Figure S2. Genetic interactions of *whi5Δ* with components of the MBF complex. Related to Figure 1.

(A) Serial dilutions of wild type, *whi5Δ*, *res1Δ*, *res1Δ whi5Δ*, *rep2Δ*, and *rep2Δ whi5Δ* cultures were spotted onto YES, MM and MMPhe plates and incubated for 4 days at 25 ºC. **(B)** Wild type, *whi5Δ*, *res1Δ*, and *res1Δ whi5Δ* strains were grown in MMPhe for 20 hours at 32 ºC. DIC images (upper panels) and microscopy cell size analysis (bottom panel) are shown. For microscopy analysis of cell size, the length of at least 300 calcofluor-stained cells of each strain from one experiment was determined. Data are represented with Tukey box-and-whisker plots. *P*-values were calculated using the Kruskal-Wallis test (one-way, non-parametric ANOVA) with Dunn's correction for multiple comparisons. **(C)** Wild type, *whi5Δ*, *rep2Δ*, and *rep2Δ whi5Δ* strains were grown in MMPhe for 20 hours at 32 ºC. DIC images (upper panels) and microscopy cell size analysis (bottom panel). At least 300 cells were measured and represented with Tukey box-and-whisker plots. Statistical significance was determined with Kruskal-Wallis test (one-way, non-parametric ANOVA) with Dunn's correction for multiple comparisons. **(D)** Wild type, *whi5Δ*, *cdc10-129*, and *cdc10-129 whi5Δ* strains were grown in MMPhe for 20 hours at 30ºC (semirestrictive temperature for *cdc10-129*). Upper panels: DIC images. Bottom panel: Quantification of cell size. At least 300 calcofluor-stained cells of each strain were measured. Results from one representative experiment are represented with Tukey box-and-whisker plots, and statistical significance was assessed using the Kruskal-Wallis test with Dunn's correction. **(E)** Analysis of cell size of wild type, *whi5Δ*, *res2Δ*, and *res2Δ whi5Δ* strains. Cells were grown in MMPhe for 24 hours at 32ºC. Upper panels: DIC images. Bottom panel: Quantification of cell size. The length of at least 300 calcofluor-stained cells of each strain were measured. Results from one representative experiment are shown. p-values were calculated with Kruskal-Wallis test with Dunn's correction for multiple comparisons. **(F)** Analysis of Rad52-YFP foci in wild type, *whi5Δ*, *rep2Δ*, and *rep2Δ whi5Δ* strains grown in MMPhe for 20 hours at 32 ºC. Upper panel: Fluorescence images of Rad52-YFP foci. Overlays with DIC images are shown. Bottom panel: Percentage of cells with one (light grey) or more than one nuclear focus (white) of Rad52-YFP. Data represent the means and standard deviations of two independent experiments. At least 500 cells were analysed per strain in each experimental replica. Scale bars: 10 µm.

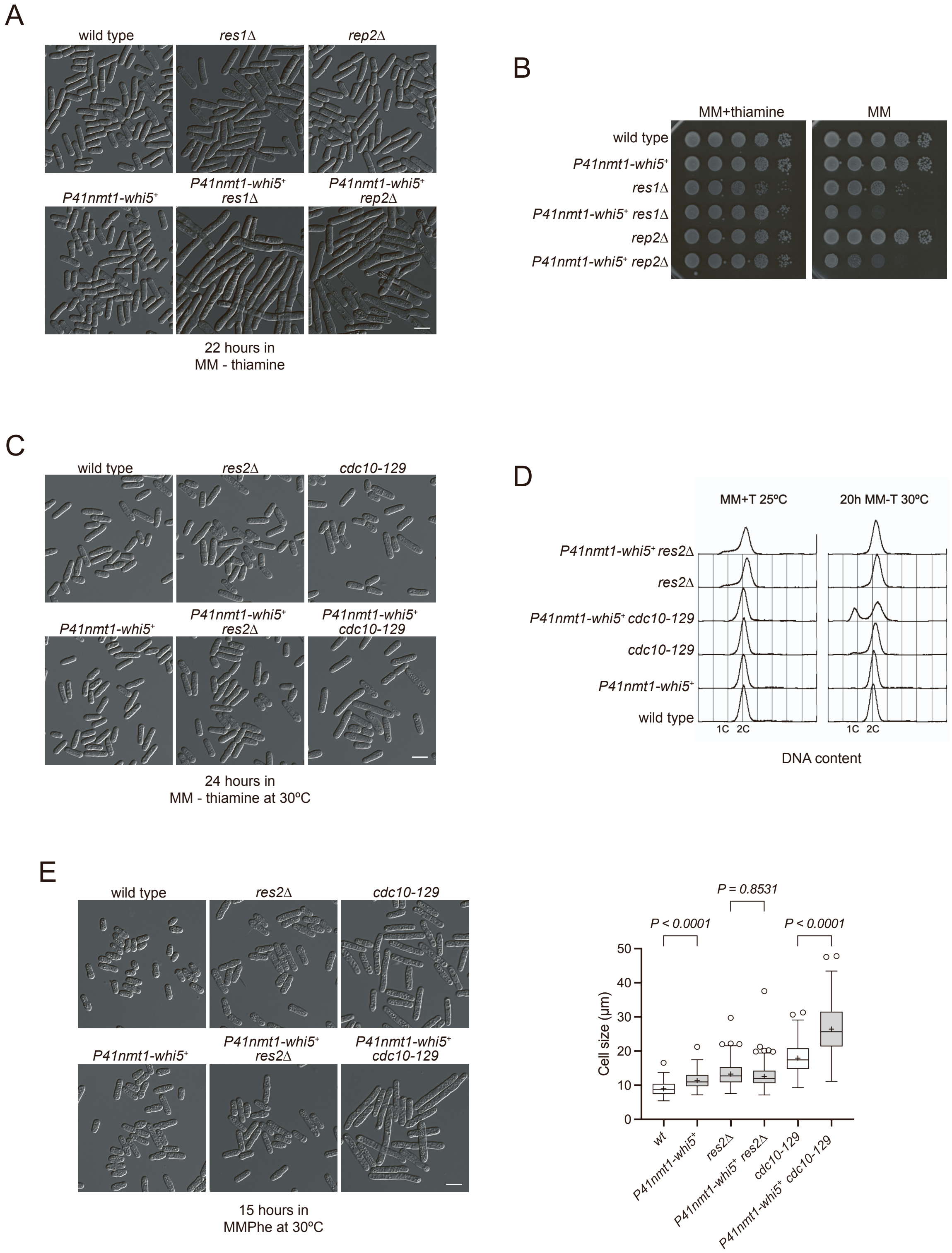
A
wild type
res1Δ
rep2Δ
P41nmt1-whi5+
P41nmt1-whi5+ res1Δ
P41nmt1-whi5+ rep2Δ
22 hours in MM - thiamine
B
MM+thiamine
MM
wild type
P41nmt1-whi5+
res1Δ
P41nmt1-whi5+ res1Δ
rep2Δ
P41nmt1-whi5+ rep2Δ
C
wild type
res2Δ
cdc10-129
P41nmt1-whi5+
P41nmt1-whi5+ res2Δ
P41nmt1-whi5+ cdc10-129
24 hours in MM - thiamine at 30ºC
D
MM+T 25ºC
20h MM-T 30ºC
P41nmt1-whi5+ res2Δ
res2Δ
P41nmt1-whi5+ cdc10-129
cdc10-129
P41nmt1-whi5+
wild type
1C 2C
1C 2C
DNA content
E
wild type
res2Δ
cdc10-129
P41nmt1-whi5+
P41nmt1-whi5+ res2Δ
P41nmt1-whi5+ cdc10-129
15 hours in MMPhe at 30ºC
P = 0.8531
P < 0.0001
P < 0.0001
Cell size (μm)
50
40
30
20
10
0
wt
P41nmt1-whi5+
res2Δ
P41nmt1-whi5+ res2Δ
cdc10-129
P41nmt1-whi5+ cdc10-129

Figure S3. Genetic interactions of *P41nmt1-whi5⁺* with components of the MBF complex. Related to Figure 1.

(A) Wild type, *res1Δ*, *rep2Δ*, *P41nmt1-whi5⁺*, *res1Δ P41nmt1-whi5⁺* and *rep2Δ P41nmt1-whi5⁺* were grown in MM+T, washed in MM to induce the expression of *whi5⁺* from the *nmt1* promoter, and incubated in MM without thiamine for 22 hours at 32 ºC. Representative DIC images are shown. **(B)** Ten-fold serial dilutions of strains shown in (A) were spotted onto MM and MM+T plates and incubated at 32 ºC for 3 days. **(C)** DIC images of wild type, *cdc10-129*, *res2Δ*, *P41nmt1-whi5⁺*, *cdc10-129 P41nmt1-whi5⁺* and *res2Δ P41nmt1-whi5⁺* cells grown in MM without thiamine for 24 hours at 30ºC (semirestrictive temperature for *cdc10-129*). **(D)** Flow cytometry of strains shown in (C) after 20 hours of growth in minimal medium with thiamine (*nmt1* promoter OFF; left panel) or without thiamine (*nmt1* promoter ON; right panel). **(E)** Wild type, *cdc10-129*, *res2Δ*, *P41nmt1-whi5⁺*, *cdc10-129 P41nmt1-whi5⁺* and *res2Δ P41nmt1-whi5⁺* cells were grown in minimal medium without thiamine for 24 hours at 25ºC to induce *whi5⁺* expression, and then transferred to MMPhe for 15 hours at 30ºC (semirestrictive temperature for *cdc10-129*). Left panels: Representative DIC images. Righ panel: Cell size quantification. Results from one representative experiment are shown. Data were represented with Tukey box-and-whisker plots. At least 300 cells from each strain were measured. Statistical analysis: Kruskal-Wallis test with Dunn's correction. Scale bars: 10 µm.

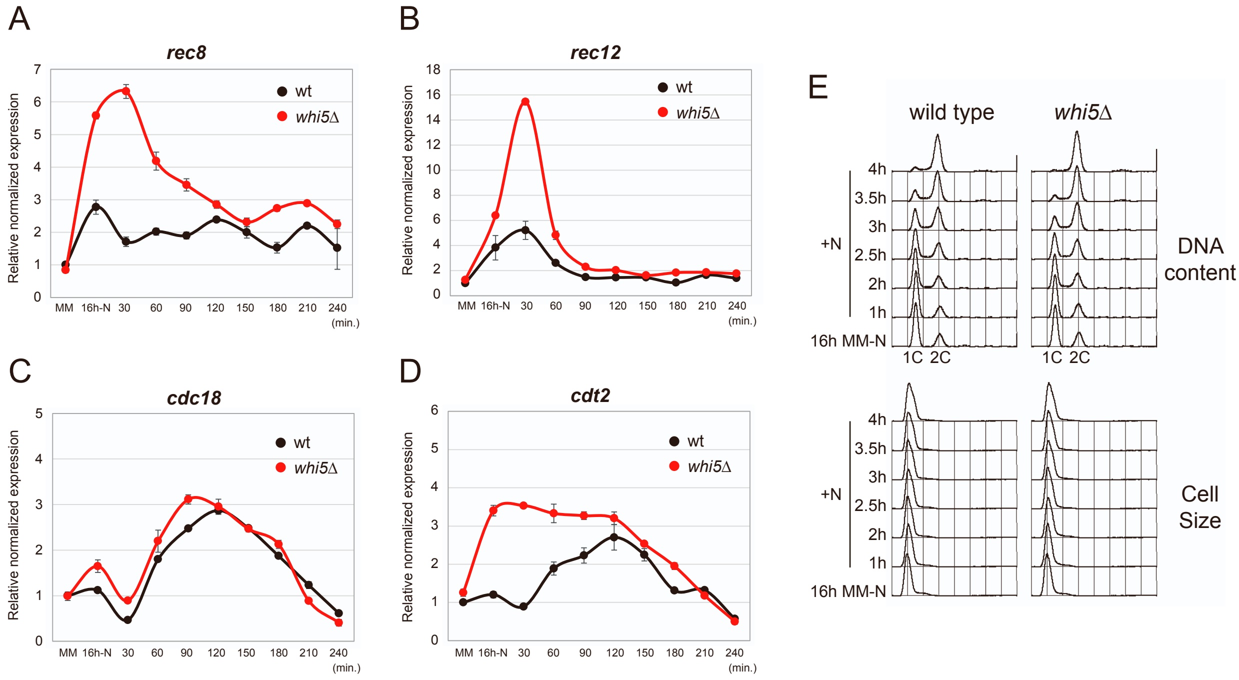

Figure S4. Expression of meiotic and S-phase genes at exit from quiescence. Related to Figure 2.

Wild-type and *whi5*$^{+}$-deleted cells were arrested in G1 by nitrogen starvation and then released by addition of NH_4Cl. The nitrogen starvation block was performed at 25 ºC and the block release at 32 ºC. Samples were taken at the indicated time points and mRNA levels of *rec8*$^{+}$ **(A)**, *rec12*$^{+}$**(B)**, *cdc18*$^{+}$**(C)** and *cdt2*$^{+}$**(D)** were determined by qPCR. The experiment was repeated twice with similar results. A representative experiment is shown. **(E)** To monitor cell cycle progression, flow cytometry analysis of DNA content and cell size was performed at the indicated time points.

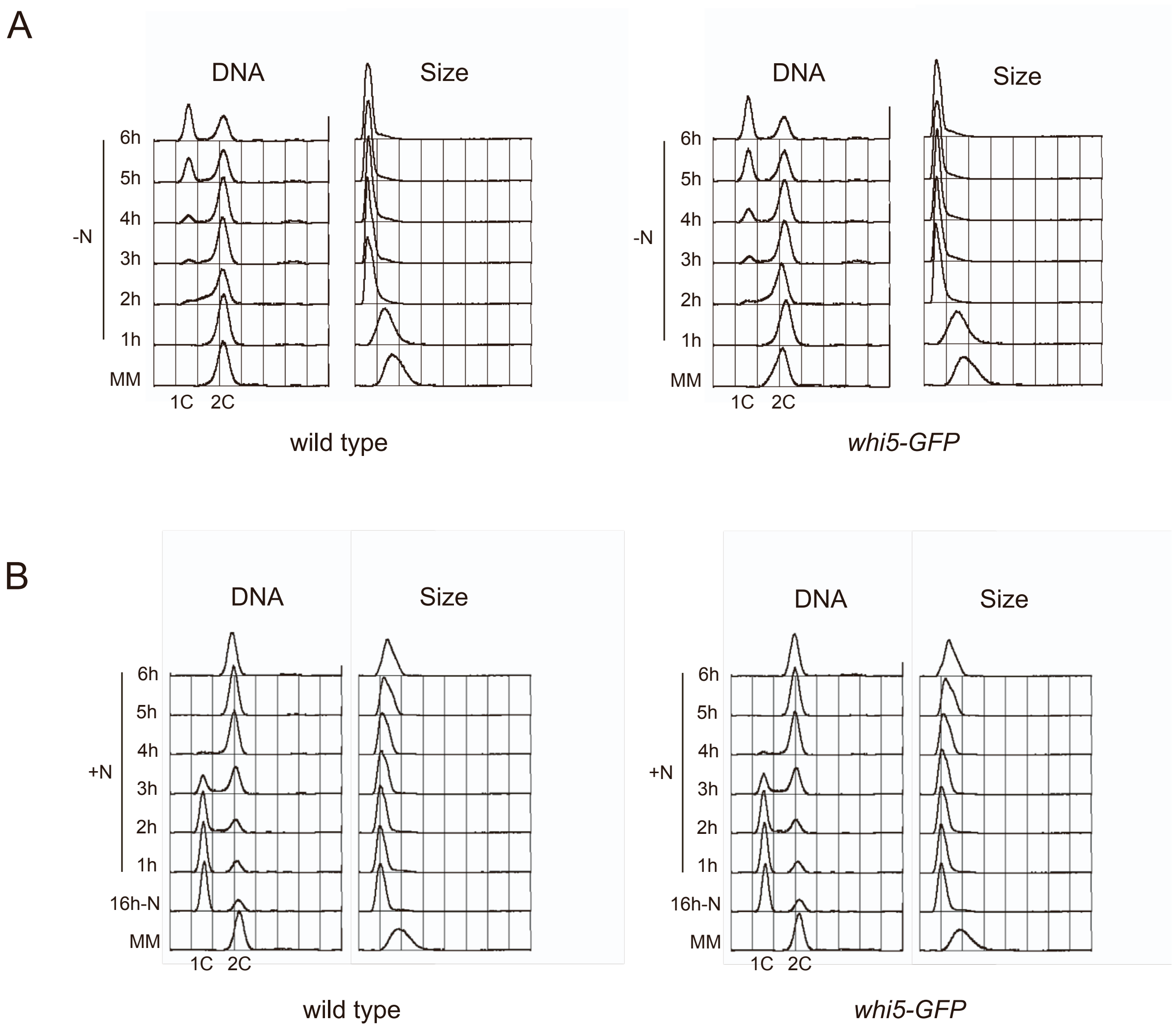

Figure S5. Flow cytometry analysis of wild type and *whi5-GFP* cells at entry into and at exit from quiescence. Related to Figure 3.

(A) Flow cytometry analysis of DNA content and cell size in *whi5-GFP* and wild-type cells at entry into quiescence. Cells were grown in MM at 25 ºC, washed twice in MM-N and transferred to MM-N at 25 ºC. Samples were taken at the indicated time points. Cell cycle arrest in G1 begins at 2 hours after the shift to MM-N. **(B)** Flow cytometry analysis in *whi5-GFP* and wild-type cells at exit from quiescence. Cells were grown in MM at 25 ºC, washed twice in MM-N, and transferred to MM-N at 25 ºC for 16 hours. Cell cycle re-entry was induced by addition of NH_4Cl. Cells were shifted to 32 ºC and samples were taken at the indicated time points for FACS analysis. The S-phase completes between 2-4 hours. The Whi5-GFP fusion did not accelerate S-phase at exit from quiescence, indicating that the C-terminal fusion of the GFP epitope did not significantly affect *whi5*$^+$ function.

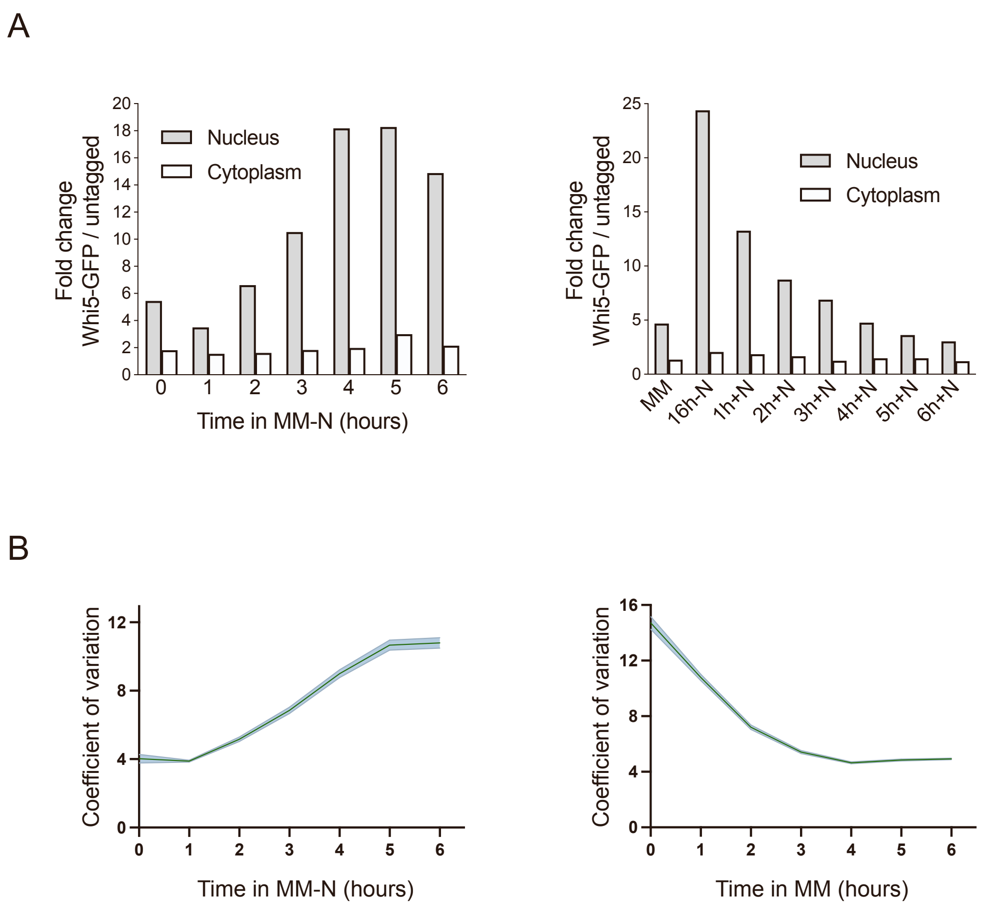

Figure S6. Quantification of Whi5-GFP in cells during entry into and at exit from quiescence. Related to Figure 3.

(A) Fluorescence intensity in the nucleus and cytoplasm was calculated at entry into quiescence (left panel; from cells shown in Figure 3A) and at exit from quiescence (right panel; from cells shown in Figure 3B). Areas from the nucleus and the cytoplasm from more than 50 cells were manually selected, and mean fluorescence intensities were calculated. The ratio between the fluorescence values of the *whi5-GFP* and the untagged strains was calculated and plotted. **(B)** Coefficients of variation (CVs) of fluorescence intensity across the cell area were used as a read-out of nuclear Whi5-GFP at entry into quiescence (left panel; cells from Figure 3A) and at exit from quiescence (right panel; cells from Figure 3B). Data were obtained from 500 cells at each timepoint. Mean fluorescence intensities and standard deviations for each cell were obtained using Fiji software. Mean CVs (solid lines) and 95% confidence intervals (shaded areas) are shown.

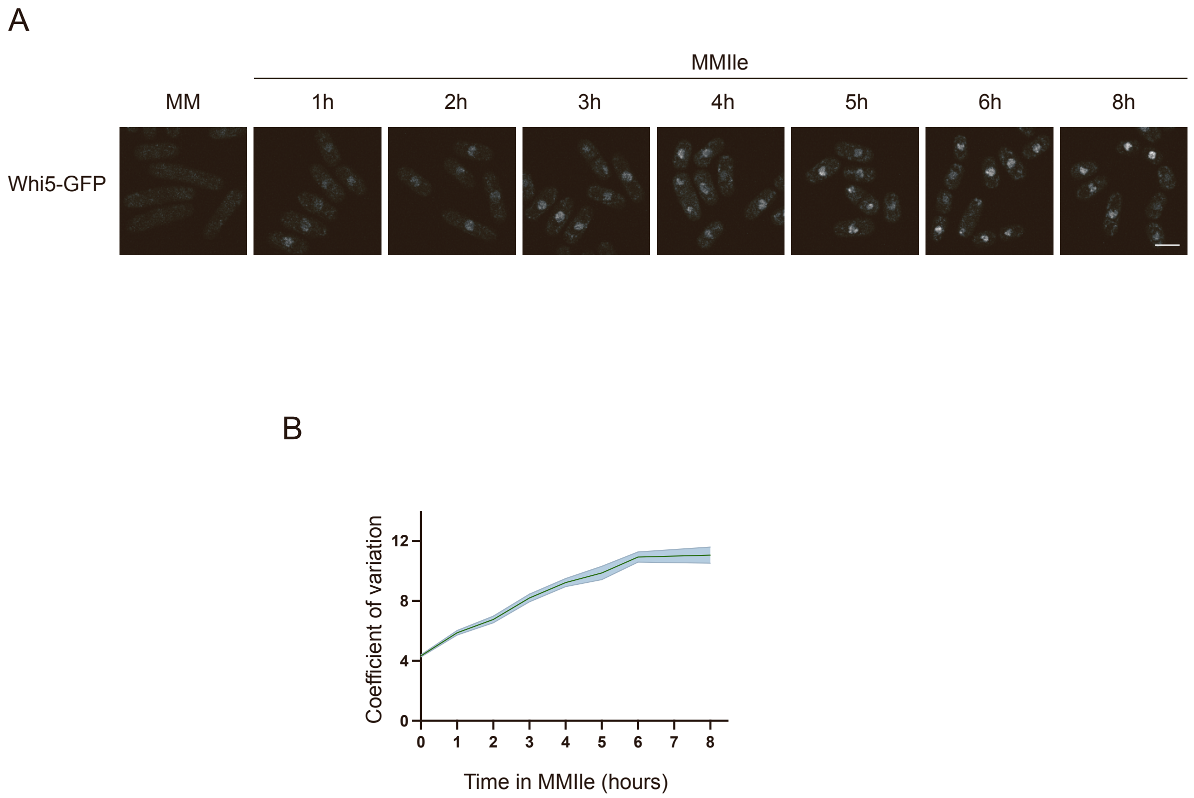

Figure S7. Whi5-GFP localises to the nucleus in nitrogen-poor medium (MMIle). Related to Figure 3.

(A) Exponentially growing cells in MM were washed twice in MMIle and cultured in MMIle at 25 ºC for 8 hours. Fluorescence images were taken at the indicated time points. Scale bar: 5 µm. **(B)** Quantification of nuclear Whi5-GFP. Coefficients of variation (CVs) of fluorescence intensity across the cell area were used as a proxy of nuclear Whi5-GFP. Data were obtained from 350 cells at each time point. Mean fluorescence intensity and standard deviations were calculated for each cell and used to determine the CVs. Solid lines indicate mean CVs and shaded areas denote 95% confidence intervals.

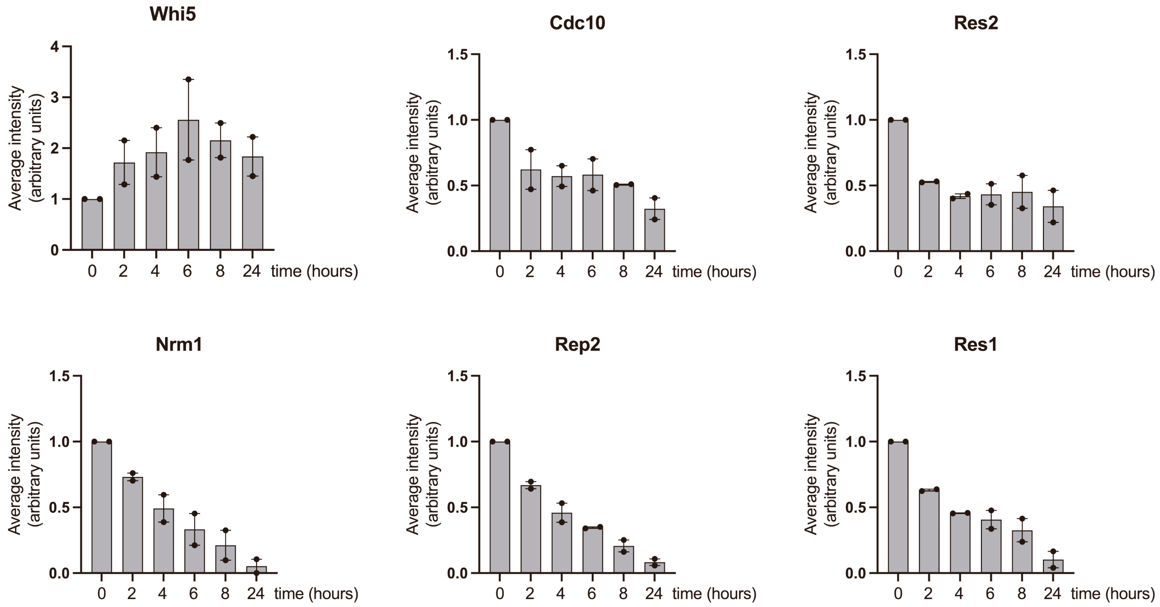

Figure S8. Protein levels of Whi5, Cdc10, Res2, Nrm1, Rep2 and Res1 during nitrogen starvation. Related to Figure 3.

Protein extracts from strains bearing Whi5-13myc, Cdc10-3HA, Res2-3HA, Nrm1-3HA, Rep2-3HA or Res1-3HA were analysed by SDS-PAGE and western blotting with anti-HA or anti-myc antibodies. Samples from time points 0, 2, 4, 6, 8 and 24 hours in MM-N were quantified with Fiji. Data represent the mean +/- S.E.M. from two biological replicates.

A

Whi5-GFP interactome

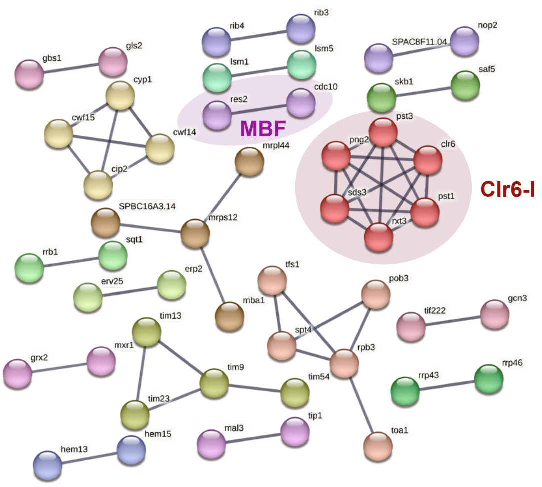

color	cluster Id	gene count	description
	Cluster 1	6	Rpd3L complex
	Cluster 2	5	RNA Polymerase II Pre-transcription Events
	Cluster 3	4	Mixed, incl. Ribosomal protein/NADH dehydrogenase domain, and Ribosomal protei…
	Cluster 4	4	post-mRNA release spliceosomal complex
	Cluster 5	4	1. Protein insertion into mitochondrial inner membrane<br>2. Translocation
	Cluster 6	2	emp24/gp25L/p24 family/GOLD
	Cluster 7	2	Mixed, incl. Lateral cortical node, and YchF, N-terminal
	Cluster 8	2	rrb1, sqt1
	Cluster 9	2	1. Exosome<br>2. mRNA decay by 3 to 5 exoribonuclease
	Cluster 10	2	1. mRNA decay by 5 to 3 exoribonuclease<br>2. Lsm1-7-Pat1 complex<br>3. snRNP Sm proteins
	Cluster 11	2	1. Porphyrin and chlorophyll metabolism<br>2. Heme biosynthesis
	Cluster 12	2	Riboflavin metabolism
	Cluster 13	2	Preribosome, large subunit precursor, and rRNA (cytosine) methyltransferase activity
	Cluster 14	2	1. Conserved DNA-binding domain that is found in a wide range of proteins of large bacterial and eukaryotic DNA viruses.<br>2. SBF transcription complex<br>3. ANK repeat
	Cluster 15	2	1. Nuclear migration involved in conjugation with cellular fusion<br>2. Mitotic spindle astral microtubule<br>3. Microtubule plus-end binding
	Cluster 16	2	Cellular oxidant detoxification, and Oxidoreductase activity, acting on a sulfur group…
	Cluster 17	2	1. Endoplasmic reticulum mannose trimming<br>2. Glucosidase II complex<br>3. Glucan 1,3-alpha-glucosidase activity
	Cluster 18	2	1. Recycling of eIF2:GDP<br>2. Eukaryotic translation initiation factor 2B complex

B

Cdc10-YFP interactome

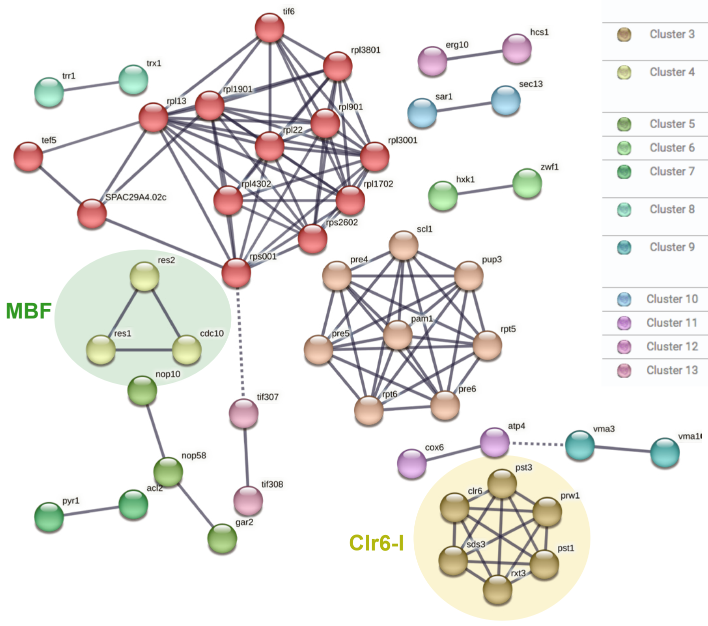

color	cluster Id	gene count	description
	Cluster 1	13	1. Ribosome<br>2. Cytoplasmic translation
	Cluster 2	8	1. Positive regulation of mitotic metaphase/anaphase transition<br>2. Proteasome<br>3. Cross-presentation of soluble exogenous antigens (endosomes)
	Cluster 3	6	1. Histone deacetylation<br>2. Rpd3L complex
	Cluster 4	3	1. SBF transcription complex<br>2. Conserved DNA-binding domain that is found in a wide range of proteins of large bacterial and eukaryotic DNA viruses.
	Cluster 5	3	sno(s)RNA-containing ribonucleoprotein complex
	Cluster 6	2	Pentose-phosphate shunt, oxidative branch
	Cluster 7	2	1. Citrate cycle (TCA cycle)<br>2. Citrate metabolic process, and Biotin-binding site
	Cluster 8	2	1. Redox-active center<br>2. Detoxification of hydrogen peroxide, and Nuclear events mediated by NFE2L2
	Cluster 9	2	1. Phagosome<br>2. Transferrin endocytosis and recycling<br>3. Vacuolar proton-transporting V-type ATPase complex
	Cluster 10	2	COPII vesicle coat
	Cluster 11	2	atp4, cox6
	Cluster 12	2	Synthesis and degradation of ketone bodies
	Cluster 13	2	Eukaryotic translation initiation factor 3 complex

Figure S9. Interactome of Whi5-GFP and Cdc10-YFP in quiescent cells. Related to Figures 5 and 6.

(A) Protein-protein interaction network comprising 166 proteins identified by mass spectrometry after immunoprecipitation of Whi5-GFP in cells cultured in MM-N for 16 hours. Proteins with an iBAQ>4000 were selected and those with iBAQ>500 in the control untagged strain were discarded. Clustering of interactomes was performed using the Markov Cluster algorithm (MCL) at the String website. **(B)** Interactome analysis of 87 proteins identified by mass spectrometry after immunoprecipitation of Cdc10-YFP from cells cultured in MM-N for 16 hours (iBAQ>3000; proteins with iBAQ>500 in the control untagged strain were discarded). As in (A), the Markov Cluster algorithm (MCL) at the String web tool was used, and the highest confidence interaction network is shown. The MBF and the Clr6-I complexes are highlighted.

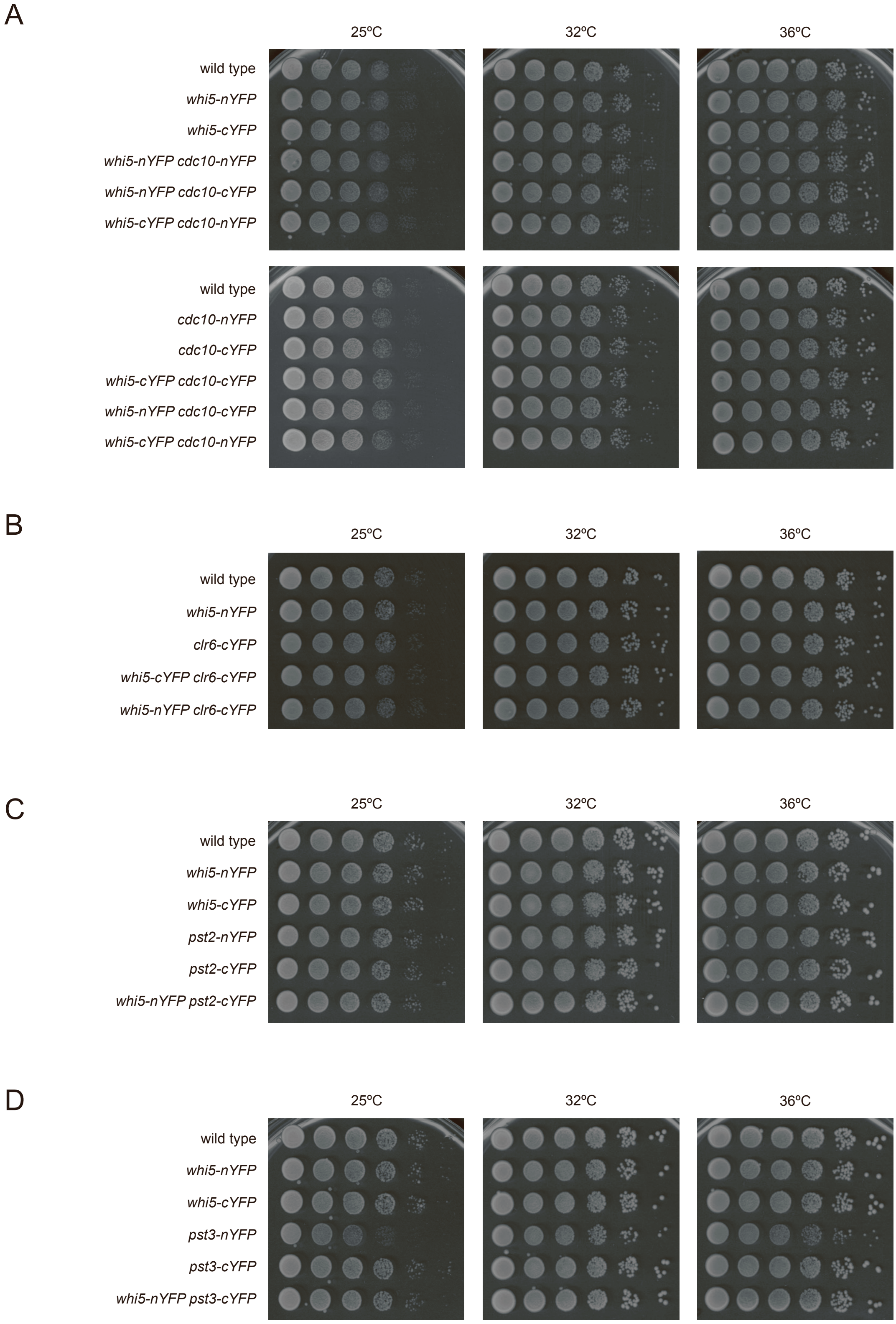

Figure S10. Viability assay of strains used to assess the interaction of Whi5 and Cdc10, Clr6, Pst2 and Pst3 by BiFC. Related to Figures 5 and 6.

Ten-fold serial dilutions of exponentially growing cells of the single and double-tagged strains used in Figures 5C and 5D **(A)**, Figure 6B **(B)** and in Figure 6D **(C** and **D)** together with an untagged control, were spotted onto YES plates and incubated at 25 ºC, 32 ºC and 36 ºC for 2-3 days.

Table S5. List of strains used in this study. Related to Key resources table.

**Strain**	**Genotype**	**Source**
**S2666**	*h^- 972*	Lab stock
**S2707**	*h- cdc10-129*	Lab stock
**S3060**	*h^- cdc10-3HA:natMX6*	This work
**S2990**	*h^- res1-3HA:natMX6*	J. Ayté
**S2991**	*h^- res2-3HA:natMX6*	J. Ayté
**S2992**	*h^- rep2-3HA:natMX6*	J. Ayté
**S2998**	*h^- nrm1-3HA:natMX6*	J. Ayté
**S3084**	*h^- nrm1-mNeonGreen:natMX6 whi5-L-mCherry:hphMX6*	This work
**S3063**	*h^- whi5-nYFP:kanMX6*	This work
**S3064**	*h^- whi5-cYFP:kanMX6*	This work
**S3065**	*h^+ cdc10-nYFP:kanMX6*	This work
**S3066**	*h^+ cdc10-cYFP:kanMX6*	This work
**S3067**	*h^- whi5-nYFP:kanMX6 cdc10-nYFP:kanMX6*	This work
**S3068**	*h^- whi5-cYFP:kanMX6 cdc10-cYFP:kanMX6*	This work
**S3069**	*h^- whi5-nYFP:kanMX6 cdc10-cYFP:kanMX6*	This work
**S3070**	*h^+ whi5-cYFP:kanMX6 cdc10-nYFP:kanMX6*	This work
**S3075**	*h^+ clr6-cYFP:kanMX6*	This work
**S3077**	*h^- whi5-nYFP:kanMX6 clr6-cYFP:kanMX6*	This work
**S3061**	*h^- cdc10-L-YFP:kanMX6*	This work

**S3062**	*h^- whi5-L-YFP:kanMX6*	This work
**S3078**	*h^+ pst2-L-YFP:kanMX6*	This work
**S3079**	*h^+ pst3-L-YFP:kanMX6*	This work
**S3080**	*h^+ pst2-cYFP:kanMX6*	This work
**S3081**	*h^+ pst3-cYFP:kanMX6*	This work
**S3082**	*h^- whi5-nYFP:kanMX6 pst2-cYFP:kanMX6*	This work
**S3083**	*h^- whi5-nYFP:kanMX6 pst3-cYFP:kanMX6*	This work
**S3058**	*h^- res1::kanMX6*	This work
**S3059**	*h^- res1::kanMX6 whi5::hphMX6*	This work
**S3091**	*h^+ rep2::kanMX4 ura4-D18 leu1-32 $ade6^-$*	Bioneer
**S3054**	*h^- rep2::kanMX4*	This work
**S3055**	*h^- rep2::kanMX4 whi5::hphMX6*	This work
**S3043**	*h^- whi5::hphMX6*	This work
**S3057**	*h^- natMX6-P81nmt1-$whi5^+$*	This work
**S3056**	*h^- natMX6-P41nmt1-$whi5^+$*	This work
**S2508**	*h^- rad52-YFP:kanMX6*	Lab stock
**S2844**	*h^- rep2::$ura4^+$ rad52-YFP:kanMX6 ura4-D18*	Lab stock
**S3048**	*h^- whi5::kanMX4 rad52-YFP:kanMX6*	This work
**S3052**	*h^- natMX6:P3nmt1-$whi5^+$ rad52-YFP:kanMX6 ura4-D18*	This work
**S3049**	*h^- whi5::hphMX4 rep2::$ura4^+$ rad52-YFP:kanMX6 ura4-D18*	This work
**S3053**	*h^- whi5-GFP:natMX6*	This work
**S3051**	*h^- whi5-13myc:natMX6*	This work
**S3088**	*h^- nrm1-3xsAID:kanMX $ura4^+$::Padh1-OsTIR1-F74A*	This work

**S3090**	*h^- nrm1-3xsAID:kanMX $ura4^+$::Padh1-OsTIR1-F74A whi5::hphMX6*	This work
**S3089**	*h^- cdc10-L-YFP:kanMX6 whi5::hphMX6*	This work
**S3095**	*h^+ kanMX6:P41nmt1-GFP-$whi5^+$ rep2::$ura4^+$ ura4-D18*	This work
**S3096**	*h^- natMX6:P41nmt1-$whi5^+$ res1::kanMX6*	This work
**S3101**	*h^- res2::kanMX6*	This work
**S3102**	*h^- res2::kanMX6 P41nmt1-$whi5^+$*	This work
**S3103**	*h^- res2::kanMX6 whi5::hphMX6*	This work
**S3104**	*h^- cdc10-129 P41nmt1-$whi5^+$*	This work
**S3105**	*h^- cdc10-129 whi5::hphMX6*	This work

Table S6. List of oligonucleotides used in this study. Related to Key resources table.

Name	Sequence	Application
**cdt2_ORF_fw**	CAGTCCTGACGGAGCAACTT	qPCR
**cdt2_ORF_rv**	GAACGCTCCCATCGTCTGAA	qPCR
**hop1_ORF_fw**	TTTTACACCGTCGCGATGGA	qPCR
**hop1_ORF_rv**	CCATTCTTTCGGGCCGTAGA	qPCR
**cnp1_ ORF_fw**	TGAAGCGTTCTTGGTTCATCTA	qPCR
**cnp1_ ORF_rv**	ACGAATCCTCCTGGCTAATTG	qPCR
**rec8_ ORF_fw**	TCGATGCCGAGTAGGGTAGA	qPCR
**rec8_ ORF_rv**	TGCGCATTCTTTTTCCATGCT	qPCR
**rec12_ ORF_fw**	ACAGCTTTTAGGCCCTCTGT	qPCR
**rec12_ ORF_rv**	GCATTCGCTGTAGCTGTTCA	qPCR
**cdc18_ ORF_fw**	TAAATTACCCACAACACCTCAAACT	qPCR
**cdc18_ ORF_rv**	ATGGAACAGGATTACATGTACGATT	qPCR
**act1_ ORF_fw**	CCTTGCTTGTTGACTGAGGCTC	qPCR
**act1_ ORF_rv**	GCAACATAAAAGGCAGGTGCAT	qPCR
**whi5-qPCR-Fw**	GCCTCCACTGCTTCAGCTAA	qPCR
**whi5-qPCR-Rv**	GAAGACTCGTGGACTTGCGA	qPCR
